# TEM1/endosialin/CD248 promotes pathologic scarring and TGF-β activity through its receptor stability in dermal fibroblasts

**DOI:** 10.1186/s12929-024-01001-0

**Published:** 2024-01-23

**Authors:** Yi-Kai Hong, Yu-Chen Lin, Tsung-Lin Cheng, Chao-Han Lai, Yi-Han Chang, Yu-Lun Huang, Chia-Yi Hung, Chen-Han Wu, Kuo-Shu Hung, Ya-Chu Ku, Yen-Ting Ho, Ming-Jer Tang, Shu-Wha Lin, Guey-Yueh Shi, John A. McGrath, Hua-Lin Wu, Chao-Kai Hsu

**Affiliations:** 1 Department of Dermatology, National Cheng Kung University Hospital, College of Medicine, National Cheng Kung University, Tainan, Taiwan; 2 International Center for Wound Repair and Regeneration (iWRR), National Cheng Kung University, Tainan, Taiwan; 3 Department of Biochemistry and Molecular Biology, College of Medicine, National Cheng Kung University, Tainan, Taiwan; 4 The Institute of Basic Medical Sciences, College of Medicine, National Cheng Kung University, Tainan, Taiwan; 5 Institute of Clinical Medicine, College of Medicine, National Cheng Kung University, Tainan, Taiwan; 6 Department of Physiology, College of Medicine, Kaohsiung Medical University, Kaohsiung, Taiwan; 7 Orthopaedic Research Center, College of Medicine, Kaohsiung Medical University Hospital, Kaohsiung Medical University, Kaohsiung, Taiwan; 8 Department of Medical Research, Kaohsiung Medical University Hospital, Kaohsiung, Taiwan; 9 Regenerative Medicine and Cell Therapy Research Center, Kaohsiung Medical University, Kaohsiung, Taiwan; 10 College of Professional Studies, National Pingtung University of Science Technology, Pingtung, Taiwan; 11 Department of Surgery, National Cheng Kung University Hospital, College of Medicine, National Cheng Kung University, Tainan, Taiwan; 12 Department of Stem Cell Therapy Science, Graduate School of Medicine, Osaka University, Suita, Osaka Japan; 13 Department of Physiology, College of Medicine, National Cheng Kung University, Tainan, Taiwan; 14 Department of Clinical Laboratory Sciences and Medical Biotechnology, National Taiwan University Hospital, Taipei, Taiwan; 15 St John’s Institute of Dermatology, School of Basic and Medical Biosciences, King’s College London, London, UK

**Keywords:** Keloid, Hypertrophic scar, TEM1/endosialin/CD248, TGF-β, Fibroblast activation

## Abstract

**Background:**

Pathologic scars, including keloids and hypertrophic scars, represent a common form of exaggerated cutaneous scarring that is difficult to prevent or treat effectively. Additionally, the pathobiology of pathologic scars remains poorly understood. We aim at investigating the impact of TEM1 (also known as endosialin or CD248), which is a glycosylated type I transmembrane protein, on development of pathologic scars.

**Methods:**

To investigate the expression of TEM1, we utilized immunofluorescence staining, Western blotting, and single-cell RNA-sequencing (scRNA-seq) techniques. We conducted in vitro cell culture experiments and an in vivo stretch-induced scar mouse model to study the involvement of TEM1 in TGF-β-mediated responses in pathologic scars.

**Results:**

The levels of the protein TEM1 are elevated in both hypertrophic scars and keloids in comparison to normal skin. A re-analysis of scRNA-seq datasets reveals that a major profibrotic subpopulation of keloid and hypertrophic scar fibroblasts greatly expresses TEM1, with expression increasing during fibroblast activation. TEM1 promotes activation, proliferation, and ECM production in human dermal fibroblasts by enhancing TGF-β1 signaling through binding with and stabilizing TGF-β receptors. Global deletion of *Tem1* markedly reduces the amount of ECM synthesis and inflammation in a scar in a mouse model of stretch-induced pathologic scarring. The intralesional administration of ontuxizumab, a humanized IgG monoclonal antibody targeting TEM1, significantly decreased both the size and collagen density of keloids.

**Conclusions:**

Our data indicate that TEM1 plays a role in pathologic scarring, with its synergistic effect on the TGF-β signaling contributing to dermal fibroblast activation. Targeting TEM1 may represent a novel therapeutic approach in reducing the morbidity of pathologic scars.

**Supplementary Information:**

The online version contains supplementary material available at 10.1186/s12929-024-01001-0.

## Background

Tissue fibrosis and scarring are constitutive parts of the wound healing process but when these are exaggerated or excessive the consequences can impose a major health and socio-economic burden [33, 94]. Currently, our understanding of the cellular and signaling events that occur when the scarring process goes awry is limited and therapeutic manipulation of the abnormal extracellular matrix (ECM) remains clinically challenging [29]. Within human skin, pathologic scarring can present as either hypertrophic scars or keloids, the etiology and management of which are presently sub-optimal [19, 85].

Keloids are characterized by aberrant activation of fibroblasts that exhibit a continuously activated myofibroblast phenotype [16]. Keloids differ from hypertrophic scars clinically in that they extend beyond the initial wound margins and fail to show spontaneous regression, and histologically by the presence of thick hyalinized eosinophilic collagen fibers (keloidal collagen bundles) [11, 84]. Excessive scarring is more prevalent in certain populations. Following surgical injury, the incidence of keloids in African, Spanish, and Asian populations ranges from 0.1% to 16%. For hypertrophic scars, the incidence is approximately 40% to 70%, with a weaker association with darker skin and genetic predisposition [30, 49, 57, 80]. Several studies have shown that scar fibroblasts, unlike normal fibroblasts (NFs), participate in scar pathogenesis by synthesizing greater amounts of ECM proteins and by enhancing abnormal responses to various molecules, such as cytokines and growth factors [59, 77].

Transforming growth factor-β1 (TGF-β1), a major profibrotic cytokine, has been found to be overexpressed in keloids and implicated in keloid pathogenesis by increasing keloid fibroblast (KF) activation [10, 51]. The binding of TGF-β1 to TGF-β receptor I/II (TGFBR1 and TGFBR2) triggers signal transduction, including the phosphorylation of SMAD2 and ERK, to induce proliferation and activation of fibroblasts into myofibroblasts [48]. An excess of activated myofibroblasts can further promote large amounts of ECM deposition in the wound, which can result in pathologic scarring.

Tumor endothelial marker 1 (TEM1), also called CD248 or endosialin, is a glycosylated type I transmembrane protein that is a member of the C-type lectin-like domain superfamily, which includes thrombomodulin and CD93 [86]. Previous studies found that TEM1 is expressed by vascular endothelial cells in several cancers [74]. However, subsequent studies convincingly demonstrated that TEM1 is expressed by fibroblasts and pericytes in tumor stroma, and not by endothelial cells [8, 17]. Not only is stromal TEM1 involved in the progression of cancers, such as abdominal tumors, breast cancer, hepatocellular carcinoma, and osteosarcoma [46, 81, 95], but it is also involved in tissue repair and scarring, including kidney fibrosis, liver fibrosis, adipocyte tissue fibrosis, atherosclerosis, and cutaneous wound healing [22, 36, 72]. In addition, TEM1 regulates fibroblast activation in diverse tissues and organs [22]. However, its precise mode of action remains unclear.

In this study, we aimed to elucidate the regulation of TEM1 in pathologic scarring as well as TGF-β-mediated myofibroblast activation, and to verify whether targeting TEM1 might prevent aberrant cellular activation in pathologic scarring. First, we demonstrate that TEM1 expression is upregulated in keloid tissues and fibroblasts, compared with normal counterparts, and show that TEM1 expression level positively correlates with several fibrogenic molecules. A re-analysis of scRNA-seq datasets from keloids reveals that the *TEM1*-expressing fibroblast subgroup is the major sub-population of KFs and we show that these cells display increased proliferation, migration, invasion, activation, and ECM production. Functional studies also illustrate that TEM1 is involved in cell proliferation, migration, and invasion of KFs. Of note, TEM1 functions by enhancing TGF-β signaling activity through post-translational stabilization and upregulation of TGF-β receptors in dermal fibroblasts. In addition, we show that global deletion of *Tem1* in mice protects against stretch-induced collagen deposition and inflammation, leading to decreased scar formation. Taken together, these results uncover a previously unrecognized role of fibroblast-expressing TEM1 in mediating TGF-β signaling activity and in promoting pathologic scar formation. The data also suggest that TEM1 could be a potential target for new therapeutic strategies to mitigate pathologic scar formation, thereby improving outcomes in individuals prone to such scarring.

## Methods

### Ethics statement and human samples

The human skin samples were obtained from the surgery room at the hospital. After the samples were obtained, they were stored in ice-cold saline to avoid drying and were instantly transported to the lab for the primary culture of fibroblasts, or the fixation of tissues, or storage at –80 °C. Keloids were diagnosed by dermatologists according to the clinical features, history, and anatomical site. All tissues assayed in this study met the pathologic criteria and features for keloids. All keloid samples were taken from active lesions with redness. The control samples were collected from patients without keloid disease who were receiving unrelated elective surgery. Information on tissue samples from the patients with normal skins or keloids is shown in Additional file 1: Table S1.

### Mouse model of traction-induced hypertrophic scarring

In *Tem1*^*lacZ/lacZ*^ mice, the single exon of *Tem1* is replaced with the *lacZ* reporter gene, as previously described [38]. Hence, *Tem1*^*lacZ/lacZ*^ mice are a *Tem1*-deficient and reporter line. *Tem1*^*lacZ/lacZ*^ mice were crossed back with C57BL/6 J mice for ten generations before conducting the experiments. After hair was removed from the back skin of male mice (8–12 weeks old) and the skin was cleaned with 70% alcohol, a 1.5-cm split wound was created using a sterile scalpel. After 6 days, the plastic device [18] was placed vertically on the split wound and traction force was induced by elongating 1 mm/day for a total of 8 mm. Therefore, scars were expanded toward both sides which are vertical to the antero-posterior axis of mice. To reduce the bias caused by sampling, we removed the upper and lower margin of the scars and cut a scar into half following the central line of the scar vertical to the antero-posterior axis of mice. One half of a scar was soaked in RNAlater solution for RNA-seq, and the other half was fixed in 10% formalin for histological staining. Thus, the cutting direction of the sections for histologic staining emanated from the central line of the scar. The front of five serial sections was used for histologic staining. We quantified the scar area of the gross view and histologic sections using image J software which was undertaken by two pathologists in a blinded manner.

### Xenograft nude mouse model of keloid

A punch biopsy, measuring 6 mm in diameter and 4 mm in thickness, was obtained from fresh keloid tissues. These biopsies were then sutured onto nude mice wounds, also measuring 6 mm in diameter, using 4–0 nylon sutures at four points. The sutures were removed two weeks post-surgery. Four weeks later, keloids were intralesionally injected either with 50 μl of PBS or with PBS containing 10 and 100 μg/ml of ontuxizumab. These injections were administered every three days, for a total of five times. The nodule sizes were assessed using micro-CT both before and after the injection process.

### Primary culture of human and mouse dermal fibroblasts

NHDFs (normal human dermal fibroblasts) (Lonza, Basel, Switzerland) were cultured in Dulbecco’s modified Eagle's medium (DMEM) with 10% FBS (Invitrogen, Waltham, Massachusetts, USA), 2 mM L-glutamine (Hyclone, Logan, Utah, USA), 1 mM sodium pyruvate (Hyclone), and 1X penicillin/streptomycin (Hyclone). Primary human normal and keloid fibroblasts were isolated from normal dermal skin of normal subjects without keloids and affected dermal lesion of keloid subjects respectively based on our previous publication [37]. Primary dermal fibroblasts were isolated from the normal skin of either *Tem1*^WT/WT^ or *Tem1*^lacZ/WT^ mice. Epidermal and dermal tissues were separated from the samples. The dermis was split into about 1-mm^3^ fragments using a scalpel. These fragments were explanted on a 10-cm culture dish with Dulbecco’s modified Eagle’s medium supplemented with 10% FBS, 2 mM L-glutamine, 1 mM sodium pyruvate, and 1X penicillin/streptomycin. The cells were cultured with growth medium refreshed every 3–4 days and were kept at 37 ℃ in a humidified incubator at 5% CO_2_. At confluence (passage 0), the cells were sub-cultured at a 1:3 ratio in a monolayer after the explants were removed. The 3rd– 8th passages of fibroblasts were used for the experiments.

### Creating stable cell lines overexpressing TEM1

We cloned the human TEM1 gene (GenBank NM_020404.3) into the EcoR I-Kpn I sites of the pEGFP-N1 vector, resulting in the pEGFP-hTEM1 construct. For the generation of cell lines with stable expression of either pEGFP-N1 or pEGFP-hTEM1, we introduced 1 μg of the respective DNA plasmids into 10^5^ HEK293 cells via electroporation. Using the Neon transfection system (Thermo Fisher Scientific, Waltham, Massachusetts, USA), two pulses were delivered at 1150 V for 20 s each. Post-electroporation, cells were given 24 h for recovery and then selected with 400 μg/ml G418. The successfully selected cells were harvested, lysed, and subjected to Western blotting to confirm TEM1 expression.

### RNA isolation and reverse transcription-quantitative polymerase chain reaction (RT-qPCR)

RNA was extracted by using an RNA isolation kit purchased from Real Biotech Corporation (New Taipei City, Taiwan) or QIAGEN (Hilden, Germany). Two μg total RNA was reversely transcribed into complementary DNA using 200 ng oligo (dT) _15_ primer, 1 mM deoxynucleotides, 5X reverse transcriptase buffer, 20 units RNase inhibitor, and 200 units M-MLV reverse transcriptase, purchased from Thermo Fisher Scientific. Then, 20 ng of complementary DNA was amplified and detected by real-time PCR machine IQ5 (Bio-Rad, Hercules, California, USA) with 400 nM primer (Integrated DNA Technologies, Inc., Coralville, Iowa, USA), 2X Fast Quant Green Master Mix with Low ROX (Protech Technology Enterprise, Taipei City, Taiwan). The relative levels of mRNA expression were measured using the 2^−ΔC^_T_ method, where ΔC_T_ was equal to (C_T_ of target gene – C_T_ of reference gene). Glyceraldehyde 3-phosphate dehydrogenase (GAPDH) was used as the reference gene. The primer sequences are listed in Additional file 1: Table S2.

### Bulk RNA sequencing (RNA-seq)

Total mRNA from 3 mouse scar tissues per group extracted by RNeasy mini kit (Qiagen) and reversely transcribed into cDNA. The cDNA was submitted for library preparation by TruSeq Stranded mRNA Library Prep Kit and sequencing using the Illumina NovaSeq 6000 platform with 150-bp paired-end reads (Illumina, San Diego, California, USA). The bases with low quality were removed using fastp software (version 0.20.0) [14]. Alignment was performed by hisat2 software (version 2.1.0) with a reference of GRCm38 [98]. Reads were counted by featureCounts software (version 2.0.0) [55]. Differential gene expression was performed using DESeq2 (version 1.34.0) [60]. Canonical pathways among gene clusters were analyzed by IPA software (QIAGEN) [47]. Cell types were predicted using xCell online software [6]. Cluster methods were performed by the tidyverse package (version 1.3.1) to explore the gene expression patterns [5]. The functional enrichment analysis of gene ontology (GO) terms among gene clusters was implemented with the clusterProfiler package (version 4.2.2) [92]. At least two different bioinformatics technicians conducted the analyses.

### Filtered raw data analysis of scRNA-seq

The Seurat R package (version 4.1.0) [12] was used to analyze the single-cell RNA-seq data of normal scars and keloids from a previously published paper [21]. Cells with fewer than 200 genes or more than 4,000 genes were detected, and more than 20% of mitochondrial genes were ruled out: 42,345 cells were obtained and subjected to downstream bioinformatic analyses. Sequencing reads of each gene were normalized to total UMIs in each cell to acquire normalized UMI values by the ‘‘*NormalizeData*’’ function. Following the merging of two scRNA-seq datasets, “*FindVariableFeatures*” was used to obtain the top 2000 variable genes. The batch effect of two datasets was eliminated by the “*IntegrateData*” function. Gene expression levels were scaled and centered using the “*ScaleData*” function. “*RunPCA*” was applied to carry out PCA dimensionality reduction. A shared nearest-neighbor graph between every cell was constructed by the “*FindNeighbors*” function. Cell cluster determination was performed by the “FindClusters” function at a resolution of 0.3. Dimensionality reduction by Uniform Manifold Approximation and Projection (UMAP) was performed by the ‘‘*RunUMAP*’’ function. For the clustering of cell subsets, each type was extracted independently and clustered by resolutions of 0.1 for fibroblasts. DEGs of each cluster with an averaged log_2_-fold change of 0.5 and an adjusted *P* value of 0.05 were determined by the default “*FindAllMarkers*” function. DEGs in the top 10 list of each cell population were shown by the “DoHeatmap” function. Cell types were annotated by “CreateSinglerObject” function on SingleR package (version 1.0.1) [7]. GO annotations for the biological functions of each gene cluster in each fibroblast subset were analyzed by the “*enrichGO*” function of the clusterProfiler package (version 4.2.2) [92].

### Pseudotime analysis

The Monocle2 R package (version 2.22.0) [73] was used to construct cell trajectories. The UMI matrix for each cell type processed by Seurat was used as input data for the “*newCellDataSet*” function. The 1000 most significant genes were selected as the ordering genes. Cells in the control sample were defined as the root state via the ‘‘*orderCells*’’ function. Dimensionality reduction was performed by the “*reduceDimension*” function with DDRTree methods. The “plot_cell_trajectory” and “plot_genes_in_pseudotime” functions were used to plot each group and gene expression levels along the same pseudotime trajectory.

### Co-immunoprecipitation

HNDF cultured with 10% FBS DMEM were lysed by the lysis buffer (0.1% Triton X-100, 0.1 M Tris-buffer, pH 8.1) with phosphatase inhibitors (1 mM Na_3_VO_4_ and 25 mM NaF) and protease inhibitors (Sigma-Aldrich, St. Louis, Missouri, USA). Cell lysates (1000 μg) were incubated with 1 μg anti-TEM1 antibody (18,160–1-AP; Proteintech, Cook County, Illinois, USA) or normal rabbit IgG (sc-2027; Santa Cruz Biotechnology, Dallas, Texas, USA) and 20 μL of protein A and G agarose beads (Millipore, Burlington, Massachusetts, USA) at 4 °C overnight. Immunoprecipitates were washed with lysis buffer 3 times and analyzed by Western blotting.

### Western blot analysis

Cell or tissue lysates were harvested in lysis buffer containing 150 mM NaCl, 50 mM Tris–HCl, and 0.01% NP40 with 1 mM PMSF (Sigma-Aldrich). Total protein (20 μg) was segregated by 8, 10, and 12% sodium dodecyl sulfate–polyacrylamide gel electrophoresis (Sigma-Aldrich) under reducing conditions and transferred to a polyvinylidene difluoride membrane. The membrane (Millipore) was blocked using 5% nonfat dry milk for 1 h at room temperature. The membrane was hybridized with the primary antibody overnight at 4 °C and washed by PBST before being incubated with horseradish peroxidase-conjugated secondary antibody (Invitrogen) for 2 h at room temperature. An enhanced chemiluminescence reagent (Millipore) was used to detect the signal captured by ImageQuant LAS 4000 mini (GE Healthcare Life Sciences, Chicago, Illinois, USA). The antibodies are listed in Additional file 1: Table S3.

### Immunohistochemistry and immunofluorescence staining for tissues

Skin samples for tissue staining were fixed with 4% paraformaldehyde in phosphate-buffered saline (PBS) at 4 °C for 1 day and then embedded in paraffin. The tissues were sectioned to a 6-μm thickness. Paraffin was removed by xylene (Sigma-Aldrich). Sections were soaked in descending alcohol. The tissue sections were boiled in sodium citrate (pH 6) for antigen retrieval, treated with 3.5% H_2_O_2_ (Sigma-Aldrich) in methanol, blocked using 5% goat serum for 1 h at room temperature, treated with the primary antibody overnight at 4 °C, and then incubated with the secondary antibody conjugated with either horseradish peroxidase (Invitrogen) or fluorescent dyes (Alexa Fluor 488- or 546-conjugated secondary antibodies) (Invitrogen) for 2 h at room temperature. For immunofluorescence staining, 4',6-diamidino-2-phenylindole (DAPI) was used as a nuclear counterstain. For immunohistochemistry**,** samples were further treated with 3,3'-diaminobenzidine (Dako, Glostrup Kommune, Denmark) and were then counterstained with hematoxylin (Millipore). The slides for immunohistochemistry were sealed with a coverslip using the Surgipath micromount (Leica Biosystems, Wetzlar, Germany). The slides for immunofluorescence staining were sealed using Flouromount-G (Invitrogen).

### Hematoxylin and eosin (H&E) staining and Masson’s trichrome (MT) staining

Hematoxylin and Eosin (H&E) staining and Masson’s trichrome (MT) staining (Sigma-Aldrich) were performed by the Human Biobank at the Research Center, Clinical Medicine of National Cheng Kung University Hospital.

### Picrosirius red stain

Tissue sections were treated with xylene and descending alcohol and stained with picrosirius red (Sigma-Aldrich) at room temperature for 1 h. Tissue sections were then washed with 1% acidified water and sealed by a coverslip using the Surgipath micromount (Leica).

### Proliferation assay

Fibroblasts were seeded at a density of 3000 cells per well in a 96-well plate with culture medium containing 10% FBS. WST-1 assays purchased from TaKaRa (Kusatsu City, Japan) were employed to assess cell proliferation at 24, 48, and 72 h. In TGF-β1-induced proliferation assay, after being seeded in a 96-well plate, fibroblasts were starved with a culture medium containing 1% FBS and then treated with 1% FBS DMEM containing 10 ng/ml of TGF-β1 (R&D Systems, Minneapolis, Minnesota, USA). Cell proliferative ability at 0, 24, 48, and 72 h was detected using a WST-1 assay.

### Wound healing assay

Fibroblasts (1 × 10^4^) were seeded in Cell Culture-Inserts (ibidi, Bavaria, Germany) with 70 ml of culture medium containing 10% FBS overnight. After the Culture-Insert was removed, a wound was formed. The cells were cultured at 37 °C for 24 h with culture medium containing 10% FBS after being washed with PBS. The zones were photographed by an inverted microscope (Olympus, Tokyo, Japan) at 0, 6, 12, and 24 h after the Culture-Insert was removed.

### Transmigration assay

Migration capacity was examined using a modified Boyden chamber assay in 24-well Transwell® plates (Corning Inc., New York, USA). Cells were resuspended using HyQTase (Hyclone). Cells (1 × 10^4^ cells per chamber) in 100 μl of serum-free medium were seeded into the upper compartment of the chambers, and culture medium containing 10% FBS was added to the lower chamber. After incubation for 4 h at 37 °C, the cells on the upper membrane were scrubbed off by a cotton swab, and the cells on the lower side of the membrane were fixed and stained with Liu’s solution (Handsel Technologies, Taipei City, Taiwan). The number of migrated cells on the membrane was counted by ImageJ software in five randomly selected views at 100 × magnification.

### Invasion assay

Cells (5 × 10^4^ cells per chamber) in 100 μl of serum-free medium were seeded into the upper compartment of chambers coated with decuple dilution matrigel (Corning Inc.), and culture medium containing 10% FBS was added to the lower chamber. After incubation for 16 h at 37 °C, the cells on the upper membrane were scrubbed off by a cotton swab, and the cells on the lower side of the membrane were fixed and stained with Liu’s solution. The number of invaded cells on the membrane was counted by ImageJ software in five randomly selected views at 100 × magnification.

### Statistical analysis

Data are displayed as mean ± standard error of the mean (SEM) of independent experiments. One-way or two-way analysis of variance and the Student’s t-test were applied to calculate statistical significance by GraphPad Prism 8.0 Software. Each experiment was repeated at least three times independently to assure the validity of the data. *P*-values of < 0.05 were considered significant. *, **, and *** indicate *P* < 0.05, *P* < 0.01, and *P* < 0.001, respectively.

## Results

### TEM1 expression is upregulated in scars and correlates with fibrotic genes and pathways

**Fig. 1:**
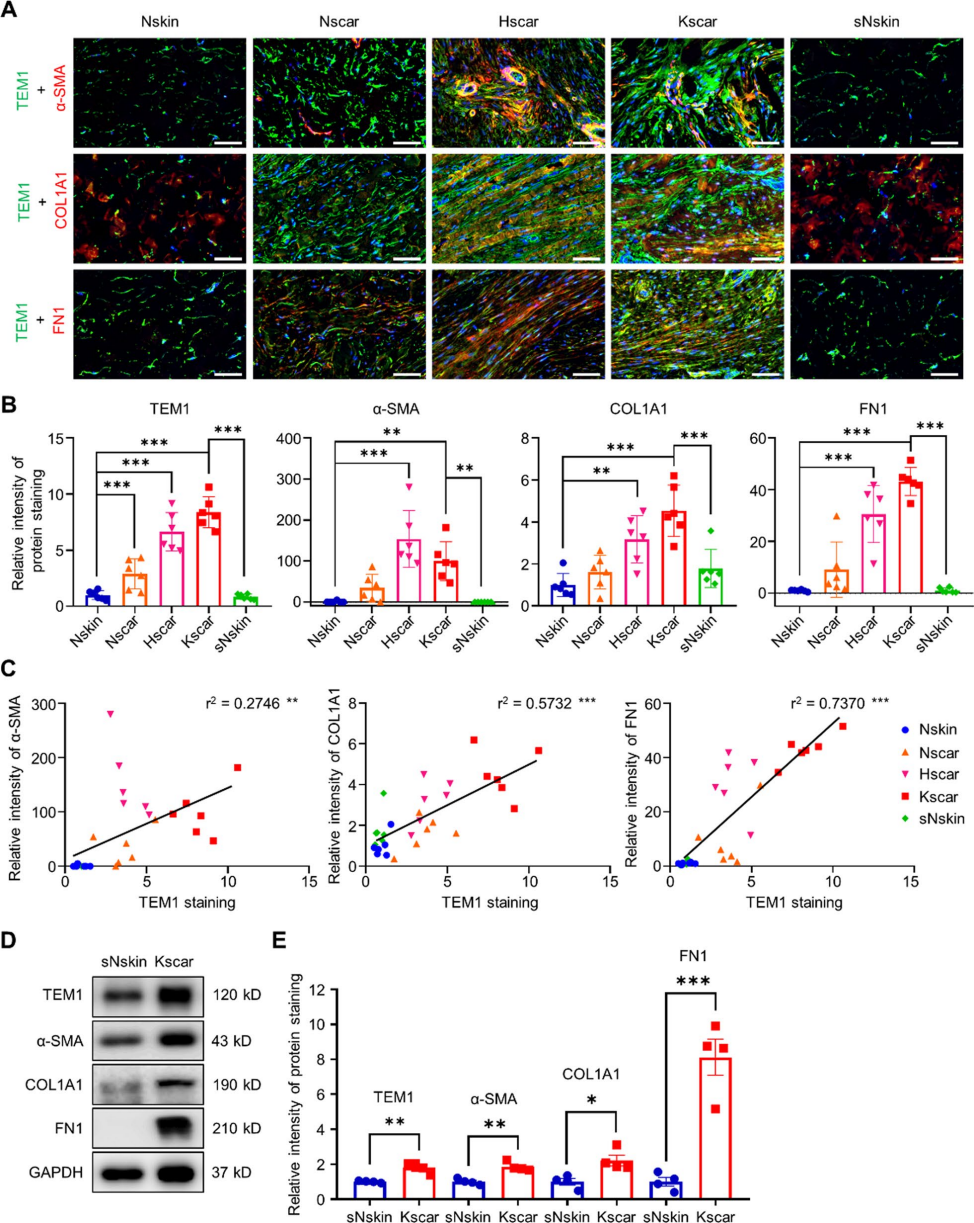
TEM1 protein expression is upregulated in keloids.** A** The tissue sections from normal skin (Nskin, n = 6), normal scar (Nscar, n = 6), hypertrophic scar (Hscar, n = 6), keloid scar (Kscar, n = 6), and surrounding normal skin of keloid (sNskin, n = 6) are immunostained for TEM1, α-SMA, COL1A1, and FN1. The nucleus is stained with DAPI. Scale bar = 50 μm. **B** The integrated density of each protein for each field in the dermis is determined using ImageJ software in a blinded manner. The random 5 fields of each samples are scanned and then quantified. The fold change of integrated intensity of TEM1 protein expression in each group is determined with reference to Nskin. **C** Pearson correlation compares TEM1 intensity with α-SMA, COL1A1, and FN1 intensity respectively in skin tissues. **D** The protein levels of TEM1, α-SMA, COL1A1, FN1, and GAPDH expression in tissues of sNskin (n = 4) and Kscar (n = 4) are analyzed using Western blotting. **E** The intensity of each protein expression relative to GAPDH is calculated based on the results of Western blotting. The fold change of relative intensity of each protein expression in keloids is compared with normal skin. Bar graphs show mean ± SEM. * *P* < 0.05, ** *P* < 0.01, *** *P* < 0.001. *P*-values are determined by one-way analysis of variance or pearson correlation analysis

To demonstrate the role of TEM1 in keloids, we analyzed the localization of TEM1 protein expression levels in normal skin (Nskin), normal scar (Nscar), hypertrophic scar (Hscar), keloid scar (Kscar), and surrounding normal skin of keloid (sNskin). TEM1 protein expression was markedly increased in Nscar, Hscar, and Kscar as compared with Nskin or sNskin, implying that upregulated TEM1 expression in human scar was induced by wounding that agreed with our previous studies in physiological wound healing of mice [36]. It is noteworthy that the expression of TEM1 in Kscar and Hscar was two-fold higher than that in Nscar, suggesting that sustained TEM1 overexpression may contribute to pathologic scar formation. In addition, TEM1 co-localized with several fibroblast-activated markers, including α-smooth muscle actin (α-SMA, an activated myofibroblast marker), as well as collagen type I alpha 1 chain (COL1A1) and fibronectin (FN1), which are major profibrotic ECM proteins, in Hscar and Kscar. The protein levels of these markers were upregulated in Hscar and Kscar, relative to the levels in Nskin or sNskin (Fig. 1A and B). Furthermore, TEM1 expression correlated positively with α-SMA (r^2^ = 0.2764), COL1A1 (r^2^ = 0.5732), and FN1 (r^2^ = 0.7370) (Fig. 1C). Using fresh protein extract from Kscar and sNskin, immunoblots showed that the protein expression levels of TEM1, α-SMA, COL1A1, and FN1 were markedly increased in Kscar, relative to sNskin (Fig. 1D and E). Re-analysis of published mRNA microarray datasets obtained from Kscar and sNskin [37] showed significant upregulation of *TEM1* mRNA in keloids, relative to normal skin. In addition, the mRNA expression levels of ECM genes including *COL1A1* and *FN1* as well as that of TGF-β-related genes such as *TGFB1*, *TGFBR1*, and *TGFBR2* were highly expressed in keloids (Additional file 1: Fig. S1). Co-expression correlation analysis of transcriptome datasets of human skin samples by Correlation AnalyzeR software [65] revealed a strong positive correlation between the gene expression levels of *TEM1* and multiple activation markers, including *ACTA2* (encoding α-SMA), *COL1A1*, *TGFBR1*, and *TGFBR2* (Additional file 1: Fig. S2A). Gene set enrichment analysis to determine the genes that correlate with *TEM1* highlighted a positive association with GO pathways including collagen-containing ECM, collagen fibril organization, regulation of ossification, TGF-β binding, and TGF-β receptor signaling (Additional file 1: Fig. S2B). The gene expression correlation of transcriptome datasets of human skin by GRNdb software [24] also showed that *TEM1* displayed a positive association with *COL1A1*, *FN1*, *TGFB1*, *TGFBR1*, and *TGFBR2* (Additional file 1: Fig. S3). Collectively, the increased expression of the TEM1 transcript and protein in pathologic scars, and the strong positive correlation of TEM1 with multiple fibrogenic genes and pathways, suggest the possible involvement of TEM1 in pathologic scar pathogenesis.

### *TEM1*-expressing fibroblasts are abundant in pathologic scars

**Fig. 2:**
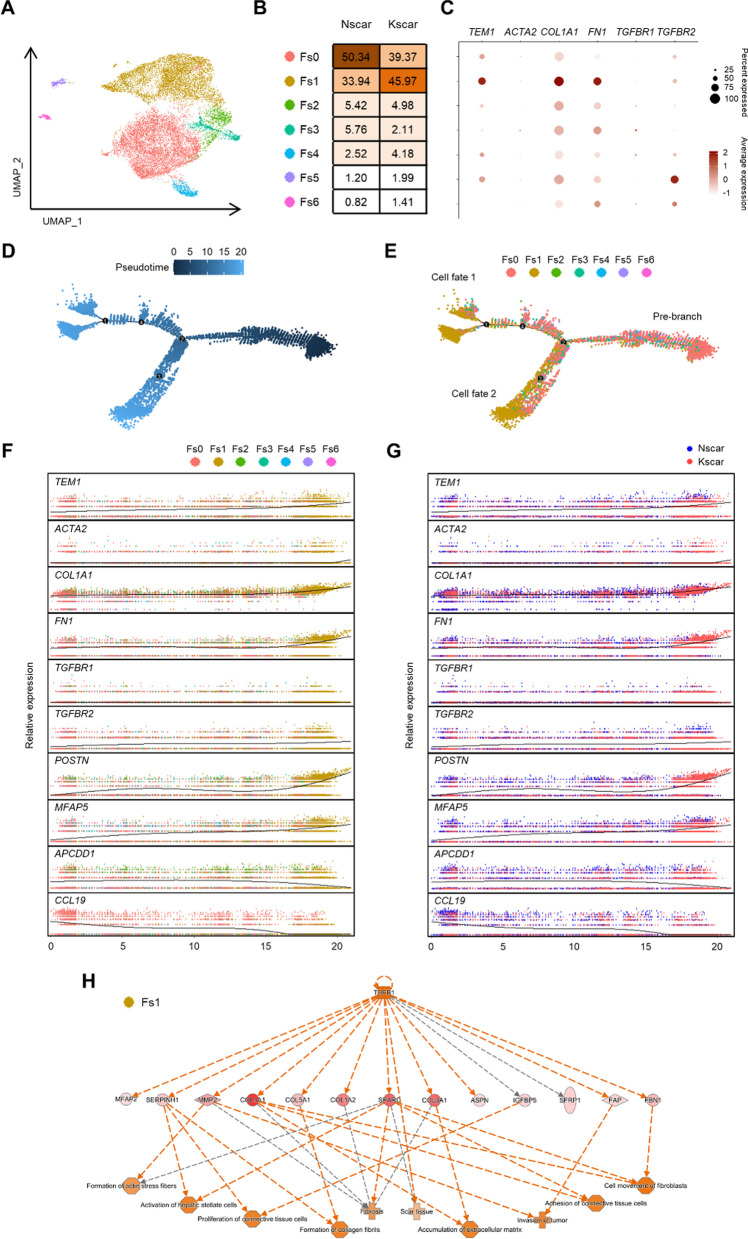
TEM1-positive fibroblasts represent the predominant and active fibroblast population in keloids at single-cell resolution. **A** The subclusters of all fibroblasts from Nscar and Kscar are further classified into 7 distinct subtypes (Fs0–Fs6). **B** Percentages of each fibroblast subgroup in normal scars and keloids are presented. **C** Sets of distinctly expressed genes in all fibroblast subsets are shown by dot plots. **D, E** The pseudo-temporal ordering of fibroblasts displays a branched trajectory. The distribution of each of the 7 subpopulations is plotted on each branch. **F, G** Kinetic plots show the relative expression pattern of 10 selected genes in all fibroblasts from Fs0 to Fs1 or from normal scars to keloids through pseudotime. **H** Upstream analysis based on DEGs in Fs1 is performed using IPA software

To explore *TEM1* gene expression in certain types of keloid cells, we re-analyzed the published single-cell RNA sequencing datasets for keloids (n = 3) and normal scars (n = 3) [21]. Unsupervised clustering by Seurat software revealed 21 cell populations including 5 fibroblast clusters (Additional file 1: Fig. S4A–C). In addition, endothelial cells and fibroblasts accounted for the major proportions of all cells which is consistent with the original publication (Additional file 1: Fig. S4D). Dot plots showed that *TEM1*, *COL1A1*, and *FN1* genes were majorly expressed in fibroblasts with a minor expression of *TGFBR1* and *TGFBR2* genes. In addition, *ACTA2* gene expression was observed mainly in smooth muscle cells, with little such expression in fibroblasts. The *TGFB1* gene was expressed primarily in myeloid cells and T cells, suggesting that both cells could be major contributors to TGF-β1 expression (Additional file 1: Fig. S4E). To further identify the expression patterns and biologic functions of TEM1 in certain fibroblast subtypes, we performed unsupervised clustering of fibroblasts and observed further heterogeneity with 7 subclusters (Fig. 2A). The transcriptomic levels of *TEM1*, as well as of *COL1A1* and *FN1*, were predominantly upregulated in the Fs1 subgroup which comprised almost half of all keloid fibroblasts (Fig. 2B and C). Pathway analysis using the GO database revealed differentially expressed genes (DEGs) within the Fs1 subgroup linked to biologic functions that include ECM organization, collagen fibril organization, and ossification, as well as to cellular response to TGF-β stimulus (Additional file 1: Fig. S5). To elucidate the dynamic expression of TEM1 during fibroblast differentiation, we used Monocle2 software to explore the pseudotime ordering of all fibroblasts (Fig. 2D). This analysis showed the pre-branch in trajectory to be majorly composed of the Fs0 subgroup, a pro-inflammatory subset (Fig. 2E and Additional file 1: Fig. S5B). Cell fates 1 and 2 predominantly consisted of the Fs1 subgroup, an ECM-producing subset (Fig. 2E). Following the pseudotime of fibroblasts from Fs0 to Fs1 (Fig. 2F) or from normal scars to keloids (Fig. 2G), the gene expression levels of *APCDD1* (a papillary dermal fibroblast marker) and *CCL19* (a pro-inflammatory marker) were observed in the beginning and were seen to decrease gradually thereafter. Interestingly, gene expression of *TEM1*, *COL1A1*, *FN1*, *TGFBR2*, *POSTN* (a mesenchymal fibroblast marker), and *MFAP5* (a reticular dermal fibroblast marker) were increasingly upregulated through pseudotime. Analysis of Fs1 by Ingenuity Pathway Analysis (IPA) software further showed that *TGFB1* plays a key upstream factor role in the proliferation, migration, invasion, and activation of fibroblasts, as well as in ECM accumulation and fibrosis (Fig. 2H). Furthermore, a re-analysis of a previously published single-cell RNA sequencing dataset for hypertrophic scars (n = 3) and normal skin (n = 3) [89] revealed that fibroblasts expressed elevated levels of *TEM1*, *ACTA2*, *COL1A1*, and *FN1* genes, along with *TGFBR1* and *TGFBR2* gene expression. Expression of the TGFB1 gene was observed in both myeloid cells and T cells, indicating that these cells may be significant sources of TGF-β1 expression. (Additional file 1: Fig. S6A–D). Unsupervised clustering of fibroblasts identified the Fs3 subgroup as having significantly upregulated transcriptomic levels of *TEM1*, *ACTA2*, *COL1A1*, *FN1*, *TGFBR1*, and *TGFBR2*. Notably, the proportion of Fs3 cells was markedly increased in hypertrophic scars (Additional file 1: Fig. S6E–G). Taken together, these results indicate that the proportion of *TEM1*-expressing fibroblasts which correlates with ECM production is enriched in keloids and hypertrophic scars, and that TEM1 could be involved in fibroblast differentiation, probably through regulating the response to TGF-β1.

### The mRNA and protein levels of TEM1 expression are augmented in keloid fibroblasts

To confirm the presence of the *TEM1*-expressing fibroblast subset in keloids identified from the scRNA-seq datasets, we performed quantitative real-time PCR and Western blotting in primary fibroblasts from keloids and normal skin. We applied quantitative real-time PCR to confirm that KFs had a higher ratio of *COL1A1* to *COL3A1* at the mRNA level in comparison to NFs, a finding which is consistent with previous data [28] (Additional file 1: Fig. S1B). The mRNA and protein levels of TEM1 expression were significantly increased in KFs relative to those of NFs (Additional file 1: Fig. S1C–E). Western blotting, to verify *TEM1*-expressing cell types, indicated that the TEM1 protein was expressed only in NFs and KFs, and not in HUVEC (endothelial cell), HaCaT (keratinocyte), or THP-1 (monocyte) cell lines (Additional file 1: Fig. S7A). To investigate the localization of TEM1 in scar tissues, double immunofluorescence staining was conducted to assess whether TEM1 co-localized with cell-specific markers for fibroblasts, endothelial cells, keratinocytes, and monocytes. In keloid tissues, TEM1 was found to co-localize with several fibroblast-activated markers, including α-SMA, COL1A1, and FN1, supporting its expression in fibroblasts within the scar tissue (Fig. 1A). Furthermore, within the context of endothelial cells, we noted that the CD31 signal exhibited a distinct circular shape defining the contours of blood vessels in the dermis. CD31 is a specific marker for endothelial cells. The TEM1 signal was observed in close proximity to the CD31-positive blood vessels, indicating that TEM1 expression may be present in perivascular cells, such as pericytes or vascular smooth muscle cells, as previously documented. [8, 34]. In the dermal compartment, TEM1 was prominently expressed in spindle-shaped fibroblasts, and it did not exhibit co-localization with the CD14 signal, which serves as a marker for monocytes. This observation further underscored the fibroblast-specific expression pattern of TEM1. Conversely, while cytokeratin 14 staining highlighted the basal and part of suprabasal keratinocytes, the TEM1 expression in the epidermis was conspicuously absent. This finding aligns with our overall conclusion that TEM1 expression is predominantly confined to spindle-shaped fibroblasts within the dermal layer (Additional file 1: Fig. S7B). Thus, the expression levels of TEM1 are upregulated predominantly in the activated fibroblasts of keloids, namely myofibroblasts.

### Global deletion of *TEM1* attenuates fibrosis and inflammation in a mouse model of pathologic scarring induced by traction force

**Fig. 3:**
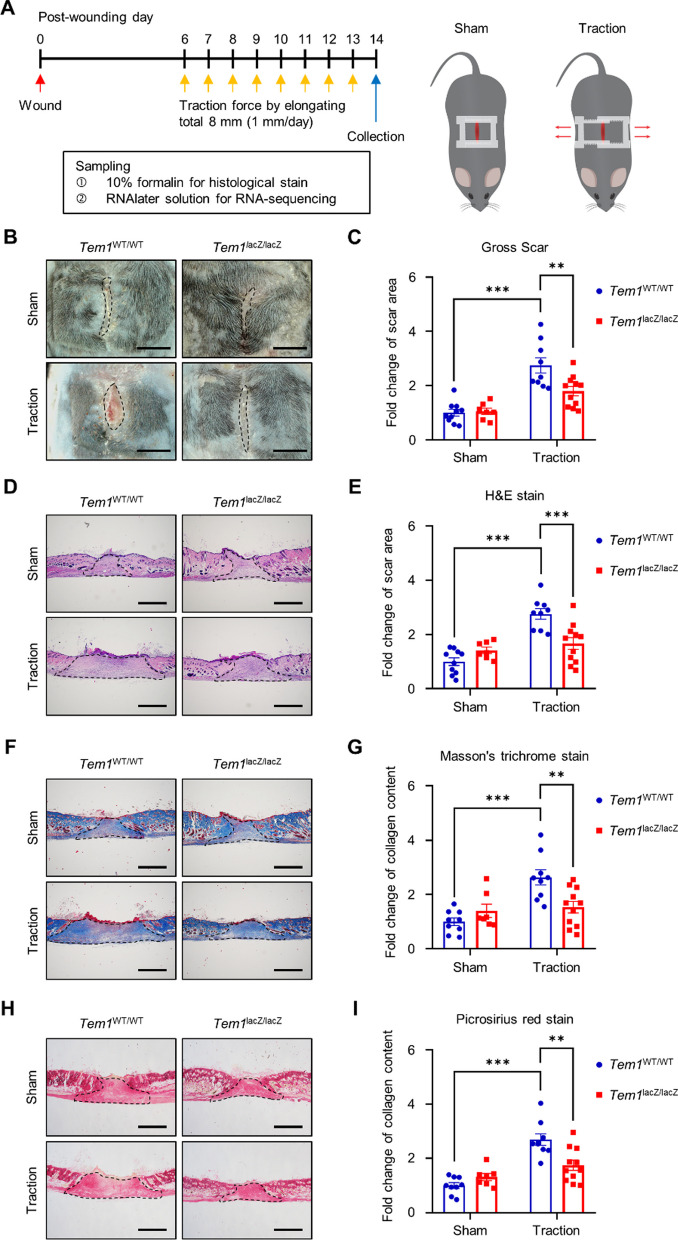
Knockout of *Tem1* in mice markedly reduces pathologic scar formation induced by mechanical stretching. **A** A flowchart for a mouse model of stretch-induced hypertrophic scarring. In brief, on post-wounding day 6, traction force in the traction group (n = 11 for *Tem1*^*WT/WT*^ mice and 13 for *Tem1*^*lacZ/lacZ*^ mice) is induced by 1 mm/day of elongation using a plastic device, for a total of 8 mm. The non-stretched scar with the plastic device is the sham control (n = 10 for *Tem1*^*WT/WT*^ mice and 7 for *Tem1*^*lacZ/lacZ*^ mice). **B, C** The gross scar in the *Tem1*^*WT/WT*^ and *Tem1*^*lacZ/lacZ*^ mice with or without traction force is captured by dissecting microscope and then quantified by ImageJ. Scale bar = 5 mm. **D, E** The scar tissues of the *Tem1*^*WT/WT*^ and *Tem1*^*lacZ/lacZ*^ mice with or without traction force are stained with H&E stain for histologic observation. The scar area is quantified by ImageJ. **F–I** Integrated intensity of collagen deposition in the scar area of the *Tem1*^*WT/WT*^ and *Tem1*^*lacZ/lacZ*^ mice with or without traction force is detected using Masson’s trichrome stain (**F, G**) and picrosirius red stain (**H, I**) and is quantified using ImageJ in a blinded manner. Scale bar = 1 mm. Bar graphs show mean ± SEM. ** *P* < 0.01, *** *P* < 0.001. *P*-values are determined by two-way analysis of variance

Since TEM1 expression is elevated in pathologic scar and correlated with myofibroblast activation, we further investigate the direct regulation of TEM1 in pathologic scar formation through in vivo studies. Notably, keloids are unique to humans and do not manifest in any other animal species. To study pathologic scarring in an animal model, we utilized a previously established mouse model of scarring that demonstrated the induction of hypertrophic scar-like fibrosis in the skin through mechanical force application [91]. Therefore, we developed this hypertrophic scar-like animal model using traction force in C57BL/6 J mice to demonstrate the crucial mechanistic effect of TEM1 on pathologic scarring. First, a split wound was created on the dorsal skin of the mouse. On post-wounding day 6, we stretched the wound by elongating the skin for a total of 8 mm (1 mm/day) using a plastic device in the traction groups. In the sham cohorts, we applied the same plastic device to the wound but did not generate any traction force (Fig. 3A). Dissecting microscopy revealed that traction force led to an increased size of the gross scar in wild-type mice (*Tem1*^*WT/WT*^). However, *Tem1*^*lacZ/lacZ*^ mice, which represent a *Tem1*-deficient line, showed smaller areas of gross scar formation induced by stretching than *Tem1*^*WT/WT*^ mice (Fig. 3B and C). In addition, the histologic results from H&E staining showed that the scar area in *Tem1*^*WT/WT*^ mice was markedly greater in the traction groups than in the sham controls. *Tem1* deletion in *Tem1*^*lacZ/lacZ*^ mice diminished traction-induced scar formation, compared with *Tem1*^*WT/WT*^ mice (Fig. 3D and E). To investigate the profibrotic role of TEM1 in scarring, we used Masson’s trichrome and picrosirius red stain to examine the impact on collagen deposition. The results showed that traction force facilitated greater collagen deposition in the scar region than in the sham groups of *Tem1*^*WT/WT*^ mice. This traction-mediated collagen synthesis was greatly reduced in the *Tem1*^*lacZ/lacZ*^ mice (Fig. 3F–I).

**Fig. 4:**
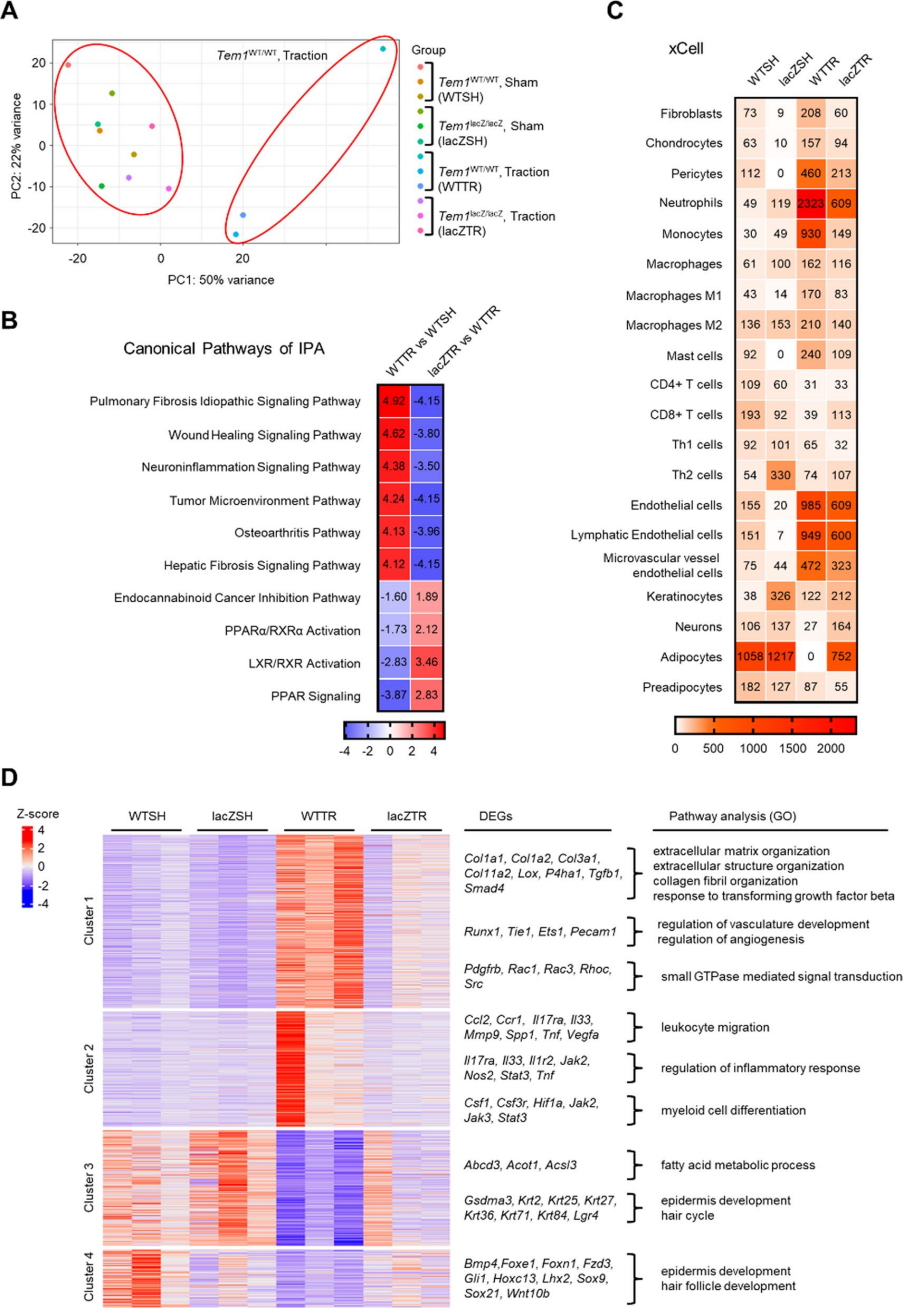
Molecular profiling by RNA-sequencing reveals that *Tem1* deficiency in mice mitigates stretch-mediated fibrosis and inflammation during scar progression. **A** The scar tissues from *Tem1*^*WT/WT*^ and *Tem1*^*lacZ/lacZ*^ mice with or without traction force (n = 3 for each group) is subjected to RNA-sequencing. Principal component analysis reveals distinct changes across four groups. **B** Canonical pathways are analyzed by IPA software following DEGs. **C** Cell type inference for each group is predicted by xCell software. **D** Cluster methods are conducted by the tidyverse package to explore gene expression patterns. The gene ontology biological processes of DEGs in each cluster are analyzed by the clusterProfiler package. *Tem1*^*WT/WT*^ mice without traction force (sham group), WTSH; *Tem1*^*lacZ/lacZ*^ mice without traction force (sham group), lacZSH; *Tem1*^*WT/WT*^ mice with traction force (traction group), WTTR; *Tem1*^*lacZ/lacZ*^ mice with traction force (traction group), lacZTR

To explore the molecular mechanism through which TEM1 regulates pathologic scarring, samples from scar areas in sham and traction groups of *Tem1*^*WT/WT*^ and *Tem1*^*lacZ/lacZ*^ mice were subjected to RNA sequencing. Principal component analysis showed that the sample clusters of *Tem1*^*WT/WT*^ mice with traction were separated from other clusters of the remaining three groups, suggesting that DEGs in *Tem1*^*WT/WT*^ mice with traction differ from those in the other groups (Fig. 4A). Canonical pathway analysis using IPA software revealed that wound healing, fibrosis, and inflammation signaling pathways were enriched in *Tem1*^*WT/WT*^ mice with traction versus the sham groups, and that *Tem1* knockout in mice abolished these traction-induced effects. When traction was applied to the wound in *Tem1*^*WT/WT*^ mice, the pathways for peroxisome proliferator-activated receptor (PPAR) signaling and endocannabinoid pathways that can inhibit the proliferation and migration in fibroblasts and cancer, respectively, were downregulated [54, 75]. Conversely, *Tem1*^*lacZ/lacZ*^ mice with traction force displayed increased enrichment of these pathways when compared with *Tem1*^*WT/WT*^ mice (Fig. 4B). To verify the regulation of TEM1 on cell populations within pathologic scarring, xCell software, for cell type enrichment analysis [6], was applied to the sham and traction groups of *Tem1*^*WT/WT*^ and *Tem1*^*lacZ/lacZ*^ mice. Traction in *Tem1*^*WT/WT*^ mice was found to increase the enrichment scores of fibroblasts, chondrocytes, pericytes, neutrophils, monocytes, and endothelial cells. Importantly, *Tem1* deficiency reduced the score of these cell types induced by traction force. In contrast, the enrichment scores for neurons and adipocytes were lower in the traction groups than in the sham groups of *Tem1*^*WT/WT*^ mice. *Tem1* knockout in mice restored the scores for both these cell types in association with decreased fibrosis in the scar region (Fig. 4C). To identify potential variation in DEGs and biologic functions regulated by TEM1, cluster methods were applied to explore whether any gene in the non-overlapping dataset exhibited a statistical interaction difference from its mean group expression level (adjusted *p*-value < 0.05) which could indicate a distinct change in gene expression (traction group relative to sham in *Tem1*^*WT/WT*^ mice) (Fig. 4D) [4]. This analysis resulted in 2,451 genes, and interaction analyses revealed 4 clusters of DEGs. Cluster 1 contained a profibrotic set of genes, including *Col1a1*, *Col1a2*, *Col3a1*, *Col11a2*, *Lox*, *P4ha1*, *Tgfb1*, *Smad4*, *Runx1, Tie1*, *Ets1*, *Pecam1*, *Pdgfrb*, *Rac1*, *Rac3*, *Rhoc*, and *Src*, that were associated with ECM organization, response to transforming growth factor beta, angiogenesis, and small GTPase signaling. Cluster 2 represented inflammatory gene sets, such as *Ccl2*, *Ccr1*, *Il17ra*, *Il33*, *Mmp9*, *Spp1*, *Tnf*, *Vegfa*, *Il17ra*, *Il33*, *Il1r2*, *Jak2*, *Nos2*, *Stat3*, *Tnf*, *Csf1*, *Csf3r*, *Hif1a*, *Jak2*, *Jak3*, and *Stat3,* which correlated with inflammatory response, leukocyte migration, and myeloid cell differentiation. DEGs in clusters 1 and 2 were upregulated in the traction groups in the *Tem1*^*WT/WT*^ mice, compared with sham groups, but the *Tem1*^*lacZ/lacZ*^ mice displayed inhibition of traction-induced expression of these genes. In cluster 3, genes including *Abcd3*, *Acot1*, and *Acsl3* were associated with the fatty acid metabolic process. Clusters 3 and 4 consisted of a gene set that included *Gsdma3*, *Krt2*, *Krt25*, *Krt27*, *Krt36*, *Krt71*, *Krt84*, *Lgr4*, *Bmp4*, *Foxe1*, *Foxn1*, *Fzd3*, *Gli1*, *Hoxc13*, *Lhx2*, *Sox9*, *Sox21*, and *Wnt10b*, which were associated with epidermal and hair follicle development. Traction in *Tem1*^*WT/WT*^ mice reduced the expression of DEGs in clusters 3 and 4, while *Tem1* deletion abolished this trend. Collectively, these data suggest that TEM1 triggers stretch-induced scar formation through ECM production and inflammatory responses.

### TEM1 is involved in the proliferation, migration, and invasion of keloid fibroblasts

**Fig. 5:**
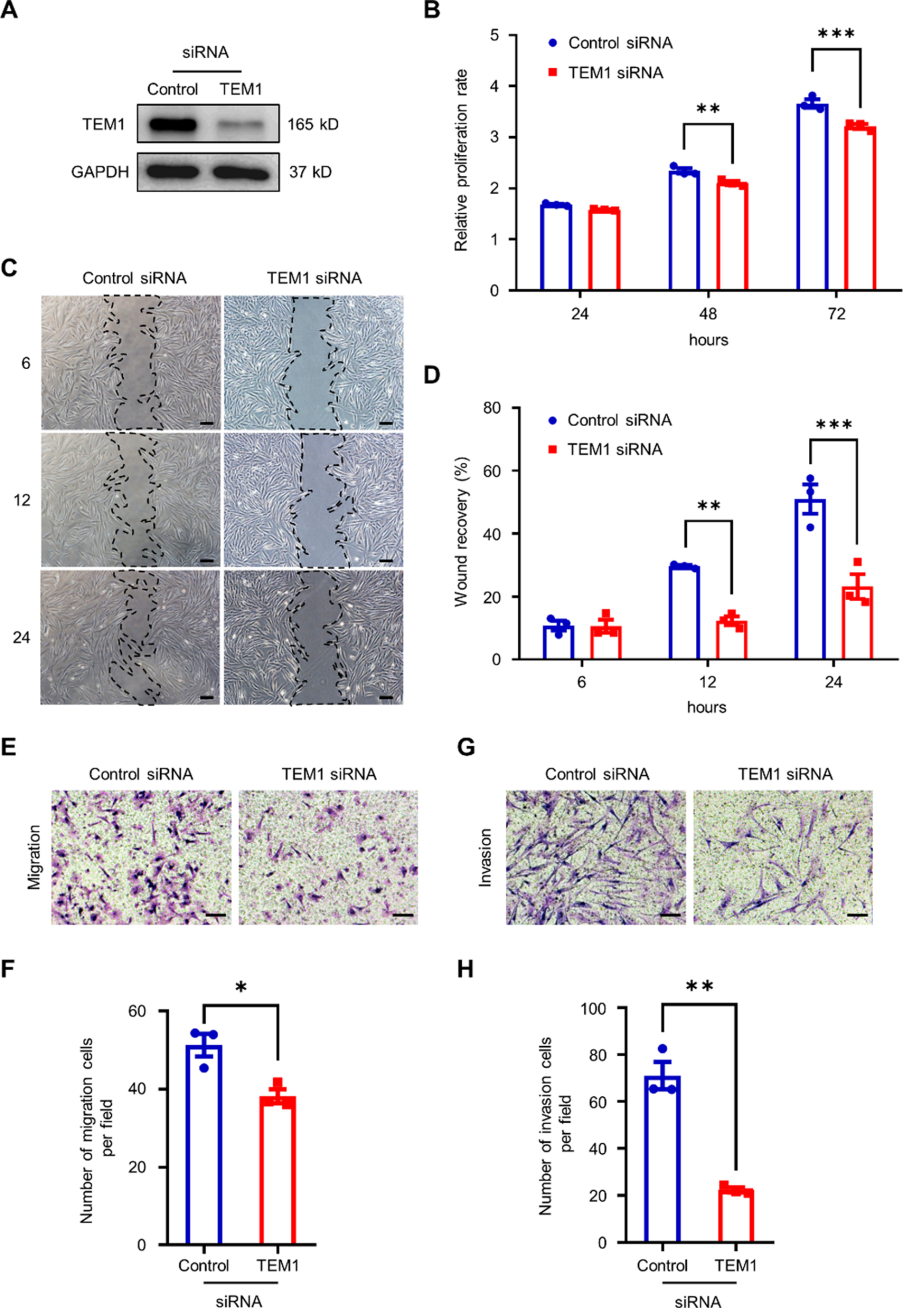
TEM1 is involved in the cell proliferation, migration, and invasion of keloid fibroblasts.** A** *TEM1*-knockdown KFs are established by transfection with *TEM1* small interfering RNA (*TEM1* siRNA). Non-target siRNA is regarded as control siRNA. The protein level of TEM1 expression is assayed by Western blotting. **B** The cell viability of fibroblasts in 10% FBS is measured by WST-1 assay. Proliferation rate at 24, 48, and 72 h relative to that at 0 h is shown. **C–F** The cell migration ability of fibroblasts is measured by wound healing assay (**C, D**) and transwell migration assay (**E, F)** with 10% FBS as the chemoattractant. Scale bar = 100 μm. **G, H** The cell invasion of fibroblasts is assayed using a Matrigel-coated transwell migration assay with 10% FBS as the chemoattractant. Scale bar = 100 μm. Bar graphs show mean ± SEM. * *P* < 0.05, ** *P* < 0.01, *** *P* < 0.001. *P*-values are determined by unpaired two-tailed Student’s *t*-test and two-way analysis of variance

We hypothesized that TEM1 can regulate the biologic functions of KFs because upregulated TEM1 expression was found in KFs (Additional file 1: Fig. S1C–E). KFs displayed a stronger capacity for proliferation, migration, and invasion than NFs (Additional file 1: Fig. S8A–E), similar to previous findings [56]. To investigate this further, small interfering RNA (siRNA) was used to knock down *TEM1* expression. The protein level of TEM1 expression in KFs was significantly reduced by the transfection of *TEM1* siRNA for 72 h, in comparison to control siRNA (Fig. 5A). Cell proliferation of KFs transfected with *TEM1* siRNA at 48 and 72 h was significantly reduced as measured by WST-1 assay (Fig. 5B). The wound recovery rate of *TEM1* siRNA-transfected KFs was also delayed in a wound healing assay (Fig. 5C and D). *TEM1* silencing in KFs significantly reduced the transmigration activity in response to 10% fetal bovine serum (FBS) gradient (Fig. 5E and F). Dermal fibroblasts primarily isolated from *Tem1*^lacZ/WT^ mice exhibited a reduced migration capability compared to those from *Tem1*^WT/WT^ mice (Additional file 1: Fig. S9A and B). In the HEK293 cell line, overexpression of TEM1 boosted migration capacity (Additional file 1: Fig. S10A–C). The invasive ability of *TEM1* siRNA-transfected KFs was also down-regulated, as demonstrated by Matrigel-coated transmigration assay in response to 10% FBS gradient (Fig. 5G and H). Overall, these results indicate that TEM1 plays a critical role in the proliferation, migration, and invasion of KFs.

### TEM1 enhances TGF-β-mediated responses of dermal fibroblasts

**Fig. 6:**
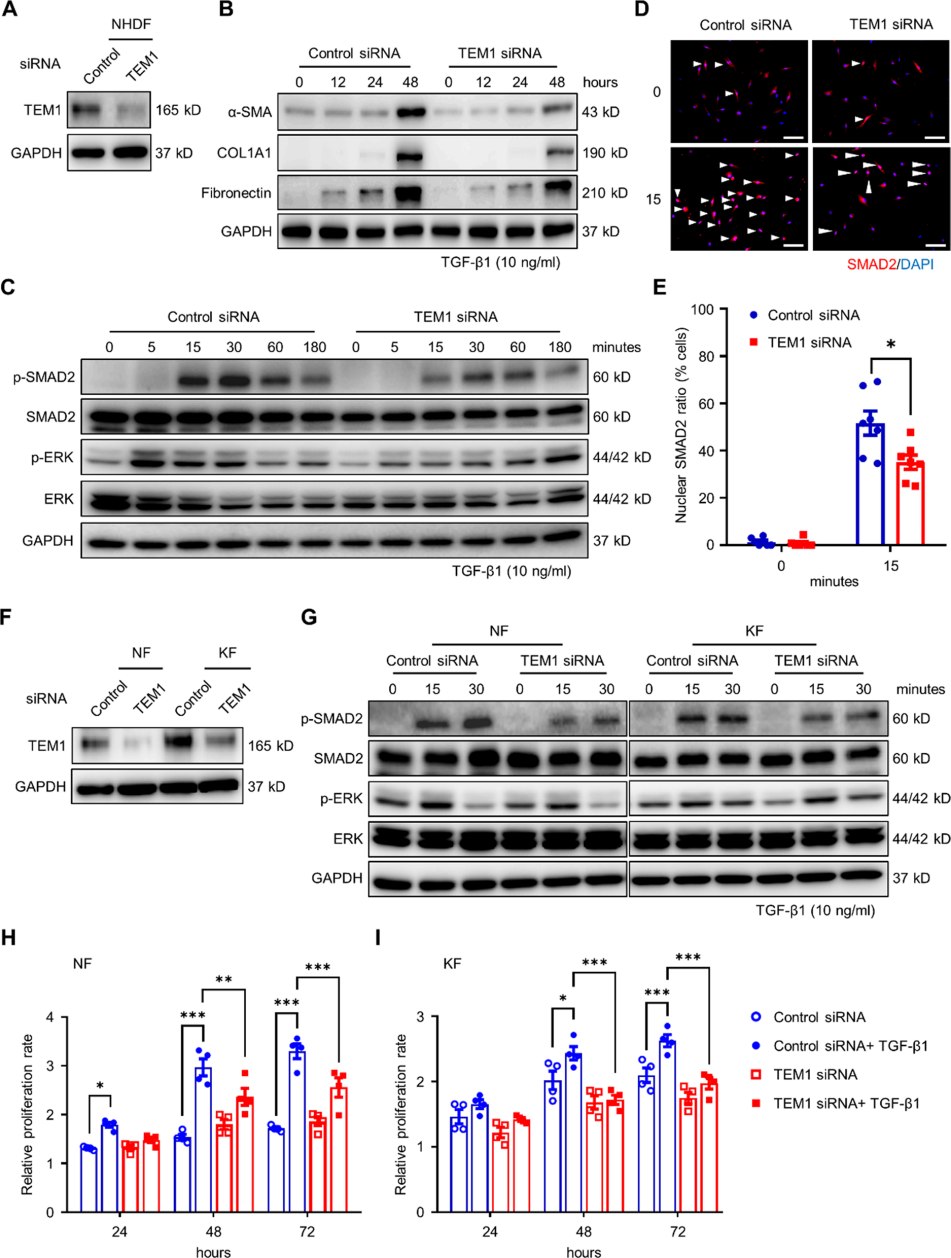
TEM1 is essential for TGF-β1-mediated activity in dermal fibroblasts.** A** The *TEM1* gene in NHDFs is knocked down by transfection with *TEM1* small interfering RNA (*TEM1* siRNA), as compared with non-target siRNA (control siRNA). The protein level of TEM1 expression is assayed using Western blotting. **B** The amount of protein expression, including α-SMA, COL1A1, FN1, and GAPDH, in NHDFs treated with TGF-β1 (10 ng/ml) for 0, 12, 24, and 48 h is analyzed using Western blotting. **C** The protein levels of p- SMAD2, SMAD2, p-ERK, ERK, and GAPDH expression in NHDFs treated with TGF-β1 (10 ng/ml) for 0, 5, 15, 30, 60, and 180 min are detected by Western blotting. **D** The nuclear translocation of SMAD2 in NHDFs after the administration of TGF-β1 (10 ng/ml) is observed by immunofluorescence. **E** The nuclear SMAD2 ratio relative to DAPI is quantified by ImageJ. Scale bar = 100 μm. **F** The *TEM1* gene in NFs and KFs is knocked down by TEM1 siRNA. The protein level of TEM1 expression is detected using Western blotting. G The protein levels of p-SMAD2, SMAD2, p-ERK, ERK, and GAPDH expression in NFs and KFs treated with TGF-β1 (10 ng/ml) for 0, 15, and 30, minutes are identified by Western blotting. **H, I** The cell viability of NFs (**H**) and KFs (**I**) in response to TGF-β1 (10 ng/ml) is measured by WST-1 assay. The proliferation at 24, 48, and 72 h relative to that at 0 h is shown. Bar graphs show mean ± SEM. * *P* < 0.05, ** *P* < 0.01, *** *P* < 0.001. *P*-values are determined by two-way analysis of variance

TGF-β1 was upregulated in keloids (Additional file 1: Fig. S1A) and was an upstream regulator of *TEM1*-positive fibroblasts in the keloid scRNA-seq datasets (Fig. 2H). In addition, *Tem1* knockout in mice decreased the expression of the traction-induced profibrotic gene cluster that is correlated with the TGF-β response pathway (Fig. 4D). We proposed that TEM1 may affect the activation of dermal fibroblasts by regulating TGF-β signal transduction. To verify this hypothesis, TEM1 protein expression was shown to be decreased in normal human dermal fibroblasts (NHDFs), NFs, and KFs by the transfection of *TEM1* siRNA (Fig. 6A and F). In NHDFs, *TEM1* knockdown significantly decreased the expression of profibrotic proteins, including α-SMA, COL1A1, and FN1, following TGF-β1 treatment for 48 h (Fig. 6B). The phosphorylation of SMAD2 and ERK (p-SMAD2 and p-ERK) that was induced by TGF-β1 stimulation was diminished in *TEM1*-silenced NHDFs (Fig. 6C). The TGF-β1-mediated phosphorylated SMAD2 can translocate from the cytoplasm to the nucleus to drive the expression of profibrotic proteins such as α-SMA, COL1A1, and FN1 [48]. Hence, *TEM1* depletion in NHDFs displayed a decrease in the TGF-β1-induced translocation of SMAD2 into the nucleus (Fig. 6D and E). In primary skin dermal fibroblasts from *Tem1*^lacZ/WT^ mice, the TGF-β1-induced nuclear translocation of SMAD2 was diminished compared to those from *Tem1*^WT/WT^ mice (Additional file 1: Fig. S9C and D). Conversely, in the HEK293 cell line, TEM1 overexpression enhanced TGF-β1-induced SMAD2 translocation (Additional file 1: Fig. S10D and E). Furthermore, the TGF-β1-driven phosphorylation of SMAD2 was also reduced in both NFs and KFs with the transfection of *TEM1* siRNA (Fig. 6G), suggesting that dermal fibroblasts (commercially obtained or from our lab) displayed a similar trend. TEM1 downregulation resulted in significantly decreased proliferative activity induced by TGF-β administration in both NFs and KFs (Fig. 6H). In sum, these results reveal that TEM1 is required for TGF-β1-induced dermal fibroblast activation, proliferation, and ECM production.

### TEM1 augments TGF-β1 signaling transduction by interacting with and stabilizing TGFBR2

**Fig. 7:**
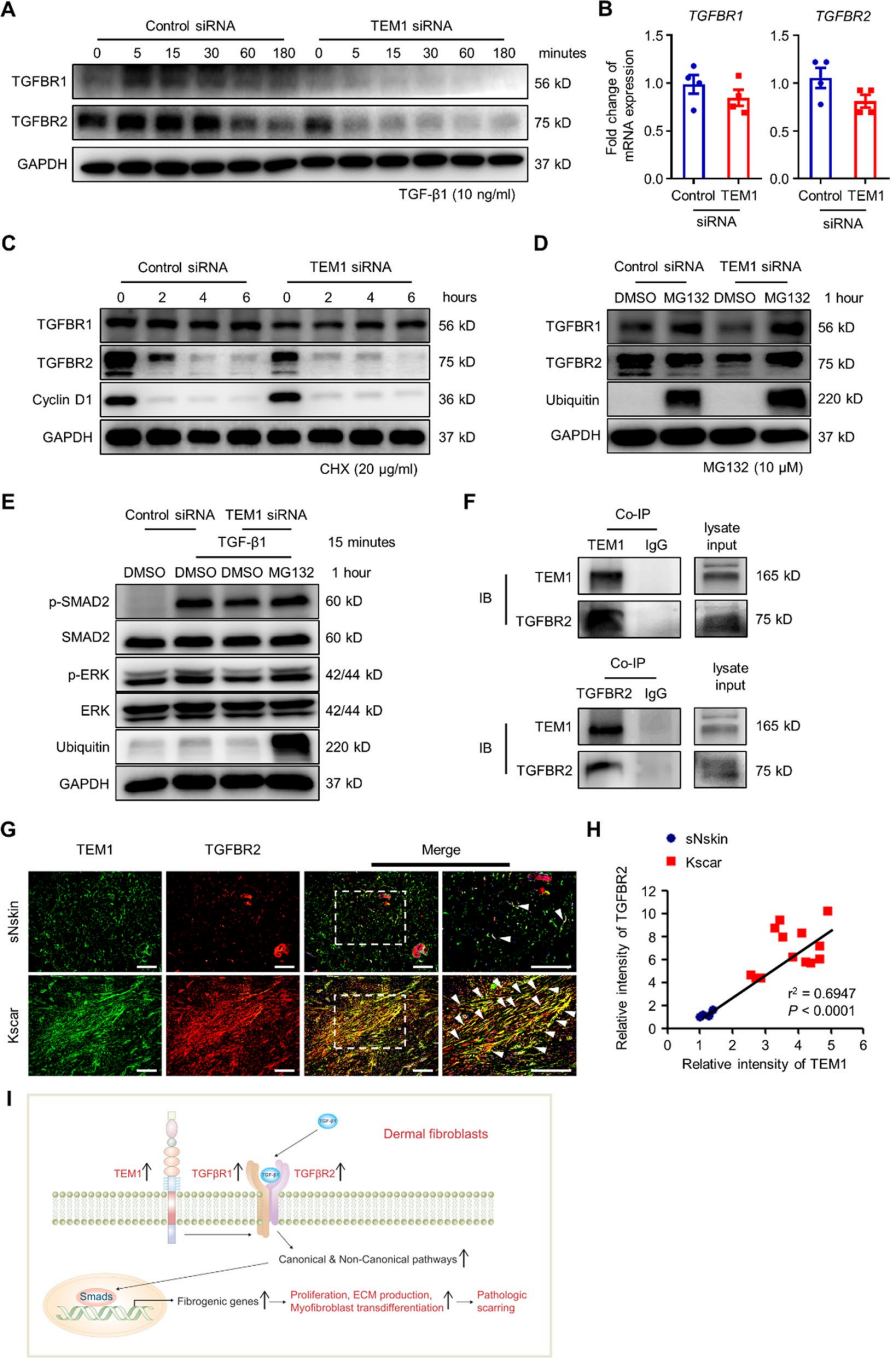
TEM1 regulates the stabilization of TGF-β receptors. **A** The protein levels of TGFBR1 and TGFBR2 expression on NHDFs transfected with TEM1 siRNA in response to TGF-β (10 ng/ml) for 0, 5, 15, 30, 60, and 180 min are determined using Western blotting. **B** The mRNA levels of *TGFBR1* and *TGFBR2* expression from the cell lysates of transfected NHDFs are detected by real-time PCR. **C** The protein levels of TGFBR1, TGFBR2, cyclin D1, and GAPDH expression on transfected NHDFs after treatment with cycloheximide (CHX) (20 μg/ml) for 0, 2, 4, and 6 h are determined by Western blotting. **D** The protein levels of TGFBR1, TGFBR2, ubiquitin, and GAPDH expression on transfected NHDFs treated with MG132 (10 μM) for 1 h are examined by Western blotting. **E** The protein levels of p- SMAD2, SMAD2, p-ERK, ERK, ubiquitin, and GAPDH expression on transfected NHDFs treated with MG132 (10 μM) for 1 h and then TGF-β1 (10 ng/ml) for 15 min are assayed by Western blotting. **F** Lysate from NHDFs cultured with 10% FBS DMEM is co-immunoprecipitated with the TEM1 antibody and immunoblotted with the TGFBR2 antibody. **G** The tissue sections from normal skins (n = 4) and keloids (n = 13) are immunostained for TEM1 and TGFBR2. The nucleus is stained with DAPI. Scale bar = 100 μm. **H** A positive correlation between TEM1 and TGFBR2 is determined by linear regression. **I** This schematic illustrates the role of TEM1 in pathologic scarring. Bar graphs show mean ± SEM

Because TGF-β1-downstream signaling pathways were impacted by TEM1, we hypothesized that TEM1 could regulate the signaling activity upstream of these kinase effectors, probably at the ligand/receptor level. *TEM1* silencing in NHDFs abolished expression of the TGFBR2 protein, but not of TGFBR1. Interestingly, TGF-β1 treatment induced the upregulation of both TGFBR1 and TGFBR2 proteins in NHDFs, which was abolished by *TEM1* knockdown (Fig. 7A). In addition, there was no change in the gene expression levels of *TGFBR1* or *TGFBR2* in *TEM1* knockdown cells (Fig. 7B). Therefore, we speculated that TEM1 could regulate the protein stability of TGFBR1 and TGFBR2 in NHDFs. To test this possibility, a cycloheximide chase assay of protein degradation was performed. Cyclin D1 protein, as a positive control, was found to gradually decrease after cycloheximide application, which is consistent with a previous study [76]. Accordingly, knockdown of *TEM1* led to the accelerated degradation of the TGFBR2 protein, but not of TGFBR1, in NHDFs treated with cycloheximide for 2 h, compared to control siRNA-treated cells (Fig. 7C). The proteasomal pathway regulates TGF-β receptor degradation and downstream signaling transduction [3, 88], and we therefore hypothesized that TEM1 regulates TGF-β receptor stability by affecting the proteasomal degradation pathway. To address this process, MG132, a proteasomal inhibitor, was used in *TEM1* siRNA-transfected NHDFs. As a positive control, we included high-molecular-weight polyubiquitinated proteins which have been reported to accumulate after MG132 treatment (Fig. 7D and E) [45]. The results showed that the reduced expression of TGFBR1 and TGFBR2 proteins in *TEM1*-deficient cells was restored after MG132 treatment for 1 h (Fig. 7D). In addition, the diminished TGF-β1-triggered phosphorylation of SMAD2 and ERK by *TEM1* knockdown was restored after MG132 administration (Fig. 7E). To sum up these results, TEM1 enhances TGF-β1-mediated signal transduction by increasing the stability of TGF-β receptors.

Since TEM1 mediated the signal transduction and receptor stability of TGF-β in dermal fibroblasts and affected post-translational TGFBR2 expression more markedly, we next considered that TEM1 might be closely associated with TGFBR2. When TEM1 protein in NHDF cell lysate was pulled down by the anti-TEM1 antibody in an immunoprecipitation experiment, a TGFBR2 protein signal was observed by Western blotting. Conversely, the TEM1 signal could also be observed when using a TGFBR2 antibody, supporting the physically close association between TEM1 and TGFBR2 (Fig. 7F). To demonstrate an association between TEM1 and TGFBR2 in vivo, we used double immunofluorescence staining in keloids and normal skins. TEM1 was found to be highly co-localized with TGFBR2 in dermal tissues from keloids and normal skin (Fig. 7G). Higher expression levels of TEM1 and TGFBR2 were detected in keloids than in normal skin; TEM1 expression correlated positively with TGFBR2 (r^2^ = 0.6947) (Fig. 7H). Taken together, these results uncover a possible association between TEM1 and TGFBR2, suggesting that TEM1 could be a modulator of TGF-β signaling.

### Ontuxizumab reduces keloid size and collagen density in xenograft nude mouse model

To investigate the role of TEM1 in vivo, we employed ontuxizumab, a humanized IgG monoclonal antibody targeting TEM1 [23, 53], in a xenograft mouse model [40, 67]. In this model, we utilized freshly obtained keloid tissues with a diameter of 6 mm and a thickness of 4 mm, acquired through punch biopsy. These keloid tissues were surgically affixed to the dorsal skin of nude mice. Subsequently, we administered intralesional injections of either PBS or ontuxizumab into the keloid tissues. After a two-week period, we harvested samples for analysis (Additional file 1: Fig. S11A). Under a dissecting microscope, we observed that the treatment with a high dose of ontuxizumab (100 μg/ml) resulted in a reduction in keloid size when compared to the PBS-treated group (Additional file 1: Fig. S11B). Micro-computed tomography further confirmed that ontuxizumab treatment at 100 μg/ml significantly reduced the volume of keloids (Additional file 1: Fig. S11C and D). Additionally, Picrosirius red staining revealed that ontuxizumab treatment at 100 μg/ml led to a reduction in collagen density within the keloid tissues (Additional file 1: Fig. S11E). We appreciate the importance of using animal models that closely resemble human keloid scarring, and our xenograft mouse model provides a valuable platform for assessing the therapeutic potential of ontuxizumab. These results significantly contribute to our understanding of TEM1-targeted therapy in the context of keloid scarring.

## Discussion

To our knowledge, this study is the first to demonstrate that TEM1, a dermal fibroblast-enriched transmembrane protein, plays a significant role in the biology of pathologic scarring. TEM1 was found to be upregulated in dermal tissues and fibroblasts from keloids, when compared with normal skin. Re-analysis of scRNA-seq datasets and immunofluorescence staining showed that TEM1 was strongly expressed by dermal fibroblasts and correlated with fibroblast activation. Mechanistic findings verified that TEM1 mediated TGF-β signaling activity through the interaction and stabilization of TGF-β receptors in dermal fibroblasts, thereby leading to excessive activation, proliferation, and ECM production of fibroblasts. Consistent with the in vitro mechanistic investigations, the global deletion of *Tem1* abrogated stretch-induced pathologic scarring by reducing TGF-β signaling, collagen deposition, and inflammation in mice. Altogether, these results reveal a crucial yet previously unknown role for TEM1 in the regulation of TGF-β signaling pathways and the pathogenesis of pathologic scarring. A schematic illustration of the molecular regulation and biologic function of TEM1 in pathologic scar formation is shown in Fig. 7I.

Unlike hypertrophic scars, keloids result from aberrant wound healing with excessive fibro-proliferation in the skin and display no spontaneous regression [30]. A special characteristic of keloids, wherein they differ from hypertrophic scars as well as from other organ fibrosis, is the sustained expansion of myofibroblasts beyond the margin of the original wound [93]. Therefore, it is likely that KFs with abnormal biologic functions play a crucial role in keloid pathogenesis. Here, our results reveal that a substantive sub-population of KFs display stronger capacities for proliferation, migration, and invasion than NFs, as previously described (Additional file 1: Fig. S8) [56]. TEM1 expression was upregulated in these KFs, relative to NFs (Additional file 1: Fig. S1C and D). Accordingly, *TEM1* silencing in KFs decreased biologic functions including cell proliferation, migration, and invasion (Fig. 5). Thus, in terms of keloid development in the skin, TEM1-expressing KFs could contribute to the progression of keloids by enhancing their hyper-responsive cell functions.

We demonstrated, in scRNA-seq datasets of hypertrophic scars and keloids, that the *TEM1* gene was mostly co-expressed with the *COL1A1* and *FN1* genes in a specific and major sub-population of fibroblasts (Fig. 2C and Additional file 1: Fig. S6G). Our immunostaining results also indicated that TEM1 expression was significantly higher in hypertrophic scars and keloids than in normal skin, as well as being associated with fibroblast activation markers such as α-SMA, COL1A1, and FN1 (Fig. 1A). Furthermore, in the mouse model of pathologic scarring, *Tem1* deficiency was found to abolish stretch-induced collagen production (Fig. 3F–I). Prior studies have shown that upregulated TEM1 expression is associated with chronic fibrotic kidney disease and liver fibrosis [79]. *Tem1*-deficient mice display the reduced mRNA levels of α-SMA and collagen expression in fibrotic tissues of kidney or liver [78, 90]. These studies, and our new data, implicate a critical role for inducible TEM1 expression in fibroblast activation and collagen deposition, such that TEM1 can be potentially regarded as a biomarker for diverse forms of tissue fibrosis. Global depletion of *Tem1* in mouse doesn’t cause any lethal embryonic defects or interfere with development or fertility [38, 68]. During embryonic periods of mouse, *Tem1* gene expression was mainly seen in vessel wall, eye, lung, liver, kidney, heart, and skin. Postnatally, *Tem1* expression decreased in most organs [38]. Importantly, Tem1 expression in adult mouse is induced during tissue injury in liver [90], lung [64], kidney [70], vessel wall [34], heart [13], and skin [36]. Therefore, targeting TEM1 expression induced by tissue damage is a possible therapeutic approach without affecting physiological functions.

TGF-β1 is the best-known cytokine trigger in keloid biogenesis [42]. TGFβ1 signaling transduction is induced by transmembrane serine/threonine kinase type II (TGFBR2) and type I (TGFBR1) receptors. First, the TGF-β1 dimer binds to TGFBR2, which then interacts with TGFBR1 and transfers phosphorylation. Phosphorylated TGFBR1 further phosphorylates Samd2/3 (canonical) and ERK (non-canonical), and both canonical and non-canonical TGFβ signaling pathways promote several profibrotic effects of TGF-β1, including fibroblast proliferation, fibroblast-to-myofibroblast transition, and ECM synthesis [27]. Although the TGF-β pathway is a potential target for therapies against pathologic scarring, few candidates that directly target TGF-β1 or its receptors have entered clinical trials [71, 97], a key limitation being that TGF-β signaling plays a crucial role in several physiological situations, including morphogenesis, embryonic development, immune regulation, and wound healing. Therefore the systemic or long-term blockage of this pathway could result in undesirable side effects, including autoimmunity, cardiovascular defects, and bone/cartilage problems, as well as chondrocyte hyperplasia [2]. Even local (injection or topical applications) therapy using TGF-β signaling inhibitors lacks specificity in treating scars and may result in adverse events, such as the development of keratoacanthomas or squamous cell carcinomas [35]. Furthermore, *Tgfb1* knockout mice display delayed wound healing with decreased re-epithelialization and granulation tissue formation [26]. Conversely, gain-of-function point mutations in the *Tgfbr1* gene in mice promote wound healing [58]. Notably, specific deletion of *Tgfbr2* in fibroblasts contributes to abnormal wound healing [63]. In addition, a human patient with Loeys–Dietz syndrome, caused by a heterozygous missense pathogenic variant in *TGFBR2,* developed delayed skin wound healing [61]. Therefore, interfering with TGF-β or its receptors directly may lack specificity in targeting pathologic scarring and may therefore not be the best therapeutic strategy to pursue. Here, we propose an alternative focus for designing new treatments for pathologic scarring, the regulation of TEM1 in TGF-β responses in dermal fibroblasts. Elevated TEM1 levels further amplify TGF-β signaling by enhancing the stability of the TGF-β receptor. Therefore, inhibiting TEM1 expression could attenuate TGF-β-mediated signaling transduction and biologic function. We found that TEM1 expression gradually increased in fibroblasts from normal scars to keloids, implying a sustained effect of TEM1 expression on pathologic scar progression. In a hypertrophic scar-like animal model induced by traction force, *Tem1* deficiency in mice led to a reduction in pathologic scar formation. Yet, in our previous study, we indeed demonstrated that *Tem1* deletion resulted in a delayed wound healing process in mice and a reduction in PDGF-mediated signaling transduction in mouse fibroblast [36]. These findings suggest that TEM1 can play a role in the early phase of normal wound healing. Moreover, in our current research, we observed that TEM1 silencing in keloid fibroblasts (KFs) led to notable effects, including a decrease in the phosphorylation of ERK and AKT, as well as a reduction in PDGFRβ protein expression induced by PDGF-BB (Additional file 1: Fig. S12). These results indicate that TEM1's involvement in both PDGF and TGF-mediated pathways could indeed contribute to pathologic scar formation. To address the concern about the potential implications of targeting TEM1, we propose that targeting TEM1 in the late phase of wound healing or once the wound has healed may represent a viable strategy for alleviating pathologic scar formation. This approach aims to mitigate the risk of interfering with physiological wound healing and other TGF-β- or PDGF-dependent functions in non-fibroblast cells. This consideration is particularly relevant due to TEM1's fibroblast-restricted expression pattern.

TGF-β1 can stimulate both TGFBR1 and TGFBR2 expression in dermal fibroblasts, indicating the positive feedback of TGFBR1 and TGFBR2 on TGF-β1-induced responses [15]. Here, we also found that TGF-β1 administration can trigger an increase in TGFBR1 and TGFBR2 protein expression in dermal fibroblasts. Most significantly, *TEM1* knockdown reduced TGF-β1-induced protein expression of both receptors (Fig. 7A). Because of the lack of any significant difference in mRNA levels between *TGFBR1* and *TGFBR2* expression in *TEM1*-knockdown fibroblasts (Fig. 7B), we speculated that TEM1 could regulate the stability of TGFBR1 and TGFBR2 proteins. This speculation was supported by the finding that *TEM1* silencing in dermal fibroblasts enhanced the protein degradation of TGFBR2 after protein synthesis was blocked by cycloheximide. In addition, TGF-β receptors are a ubiquitination target via a specific ubiquitin E3 ligase for degradation.[39, 41] The degradation of TGF-β receptors through the ubiquitin proteasome pathway turns off TGF-β signaling [87]. Interestingly, MG132 treatment, an inhibitor of the proteasomal protein degradation pathway, restored the reduced TGF-β receptor proteins and the TGF-β-induced phosphorylation of SMAD2 in *TEM1*-knockdown cells (Fig. 7D and E). Previous studies have shown that betaglycan and endoglin promote TGF-β signaling by associating with TGF-β receptors [31, 50]. In this study, we propose that TEM1 is a further essential mediator for TGF-β signal transduction. Accordingly, the association between TEM1 and TGF-β receptors was demonstrated (Fig. 7F–H). Consequently, TEM1 can facilitate TGF-β1 signaling pathways through the interaction and stabilization of TGF-β receptors.

Increasing evidence suggests that inflammatory-response dysregulation contributes to pathologic scar formation [96]. Of note, knockout of fibroblast-specific focal adhesion kinase in a mouse model of hypertrophic scar formation substantially decreased fibrosis through attenuated CCL2 signaling and inflammatory cell recruitment [91]. This result indicates the important effect of fibroblasts on both fibrosis and inflammation. Here, we identified TEM1 as a specific marker for fibroblasts (Fig. 1E and F). TEM1 deletion in a mouse model of traction-induced hypertrophic scar significantly downregulated the gene sets related to ECM synthesis as well as leukocyte recruitment (Fig. 4D). Interestingly, scRNA-seq datasets of keloids showed that a *TEM1*-expressing Fs5 subgroup with an increased proportion of fibroblasts in keloids was associated with immune functions (Fig. 2C and Additional file 1: Fig. S5). What is more, in white adipocyte and renal tissues, targeting TEM1 specifically in adipocytes or fibroblasts diminishes tissue fibrosis and inflammation [70, 72]. In atherosclerosis studies, significantly fewer macrophages were found to transmigrate towards the supernatant collected from vascular smooth muscle cells from *Tem1*-knockout mice compared to wild-type mice. That study also showed significantly decreased expression of *Ccl2* and *Ccl5* genes in isolated and stimulated vascular smooth muscle cells from the *Tem1*-knockout mice [34]. We also verified that *Tem1* deficiency in mice downregulated traction-induced *Ccl2* gene expression in pathologic scars (Fig. 4D). Therefore, these two lines of evidence indicate that TEM1 could also regulate inflammatory responses indirectly by modulating the secretion of cytokines or chemokines during tissue fibrosis.

Increased expression of TEM1 mRNA and protein can be observed in tissues and fibroblasts from keloids compared to non-keloid material (Fig. 1 and Additional file 1: Fig. S1). In addition, TEM1 expression is significantly upregulated not only in keloids but also in other fibrotic tissues [9, 70, 90]. However, currently it remains unclear how TEM1 expression can be induced initially during tissue fibrosis. A previous paper identified type I/IV collagen and fibronectin as specific ligands for TEM1 [82]. To investigate the interaction of ligand-receptor pairs, including *COL1A1*-*TEM1*, *COL4A1*-*TEM1*, and *FN1*-*TEM1*, we analyzed signal communications among all cell groups in the scRNA-seq data of keloids using CellChat [43]. The results revealed that *COL1A1* and *FN1* were predominantly secreted by C2_FB, while *COL4A1* mainly originated from C3_SMC. *TEM1* in C2_FB primarily received these signals. This finding suggests that C2_FB may interact with extracellular matrix components such as *COL1A1*, *COL4A1*, and *FN1* through *TEM1* (Additional file 1: Fig. S4F). Therefore, we can’t exclude the possibility that these interactions could create a positive feedback loop contributing to TEM1 upregulation in keloid fibroblasts. Additionally, another potential modulator of TEM1 expression in keloids could be hypoxia-induced factors (HIFs). Under hypoxic conditions, HIFs have the capacity to bind to the TEM1 promoter, thereby promoting TEM1 gene expression in human cells [69]. Furthermore, keloid tissues and fibroblasts exhibit redundant expression of HIFs. Notably, hypoxia has been demonstrated to augment TGF-β-mediated signal transduction, thereby promoting the transition of fibroblasts into myofibroblasts within keloids [52, 99]. Consequently, it is possible that hypoxia-induced TEM1 expression in keloids may contribute to fibroblast activation through a synergistic effect on TGF-β pathways, rather than exerting a direct action on fibroblasts by TEM1 itself.

TEM1 can regulate the activation of several membrane-associated signal transduction contributors such as TGF-β, platelet-derived growth factor-BB, and insulin-like growth factor 2 [36, 62, 66, 83, 90]. Hence, which specific membrane-associated signal can be modulated dominantly by TEM1 in pathologic scarring is a critical issue. Our in vitro studies with dermal fibroblasts further demonstrated that TEM1 enhanced TGF-β-mediated ECM synthesis, myofibroblast activation, and cell proliferation by affecting the phosphorylation of SMAD2 (Fig. 6). Additionally, we confirmed that suppressing TEM1 in KFs resulted in reduced phosphorylation of ERK and AKT, along with a diminished expression of the PDGFRβ protein triggered by PDGF-BB (Additional file 1: Fig. S12). These findings suggest that TEM1's participation in both the PDGF and TGF-mediated pathways may contribute to the development of pathologic scar formation. However, in the animal model resembling hypertrophic scars, we observed that the depletion of *Tem1* in mice led to a decrease in the expression of the stretch-induced profibrotic gene cluster 1 (Fig. 4D). This cluster is associated with multiple biological pathways, including TGF-β, Wnt signaling pathway, regulation of G protein-coupled receptor signaling pathway, PDGFB signaling pathway, cellular response to vascular endothelial growth factor stimulus, response to fibroblast growth factor and epidermal growth factor receptor signaling pathway (Additional file 1: Table S4). Thus, we cannot rule out the possibility that TEM1 might also influence the activation of other membrane-associated signaling pathways.

The upregulated signal of soluble TEM1 (sTEM1) was detected in heart failure patients' plasma, showing a strong correlation with cardiac fibrosis biomarkers [25]. Additionally, sTEM1 plays a role in binding to the PDGF receptor and activating the PDGF downstream signaling pathway in cardiomyocytes [13]. Our research revealed increased sTEM1 expression in conditioned medium derived from keloid tissues and fibroblasts (Additional file 1: Fig. S1F). This implies that sTEM1 may interact with TGF-β receptors, possibly enhancing TGF-β-induced activation of dermal fibroblasts in keloids. This underscores the potential of sTEM1 as a prognostic biomarker for ontuxizumab treatment, though more comprehensive studies are required. Furthermore, ontuxizumab application in keloids could reduce collagen expression, likely by disrupting feedback loops between TEM1 and ECM proteins. Given that TEM1 primarily emerges during pathologic scarring and acts as a crucial mediator in ECM production, its inhibition by ontuxizumab may provide a therapeutic pathway without affecting the physiologic functions of ECM proteins. Nonetheless, systemic administration of ontuxizumab in treating melanoma, sarcomas, and colorectal cancer has resulted in adverse effects in clinical trials [20, 32, 44]. Consequently, local administration of ontuxizumab may present a more advantageous approach for managing pathologic scar formation.

Although there are some notable differences, keloids and hypertrophic scars share several common features, including excessive production of the ECM, activation of myofibroblasts, and increased activity in the TGF-β pathway [1, 57]. ScRNA-seq analysis identified consistent expression patterns for TEM1, ACTA2, COL1A1, FN1, TGFBR1, and TGFBR2 in distinct fibroblast subsets of both keloids (Fig. 2 and Additional file 1: Fig. S4) and hypertrophic scars (Additional file 1: Fig. S6). These findings indicate the presence of fibroblast subsets with similar aberrant TGF-β signaling in both types of pathological scars. Moreover, TEM1 protein expression was markedly elevated in normal scars, hypertrophic scars, and keloids when compared to normal skin. Notably, TEM1 expression in keloids and hypertrophic scars was approximately two-fold higher than that in normal scars, suggesting that sustained TEM1 overexpression may indeed contribute to the formation of both pathological scar types (Fig. 1A and B). Our findings demonstrated that silencing TEM1 resulted in a decrease in TGF-β1-mediated collagen synthesis and fibroblast activation in dermal fibroblasts (Fig. 6). Deletion of TEM1 significantly slowed down collagen deposition and inhibited TGF-β response pathways in the scar area of the traction-induced hypertrophic scar mouse model (Fig. 3 and 4). In the xenograft nude mouse model, anti-TEM1 antibody decreased both keloid size and collagen density. Hence, we demonstrated a crucial yet previously undiscovered role for dermal fibroblast-expressing TEM1 in the TGF-β signaling and progression of pathologic scarring.

## Conclusions

Our study reveals that TEM1 amplifies pathologic scarring by enhancing TGF-β signaling. It achieves this through the post-translational stabilization of TGF-β receptors in dermal fibroblasts, leading to heightened activation, proliferation, and ECM production. Notably, TEM1 protein levels are substantially higher in hypertrophic scars and keloids compared to normal skin. This underscores TEM1's pivotal role in pathologic scar development, positioning it as a potential diagnostic indicator and a therapeutic target for addressing fibrosis.

### Supplementary Information

**Additional file 1: Fig. S1.** TEM1 is upregulated in the tissues and fibroblasts of keloids. **Fig. S2. **TEM1 is correlated with TGF-β pathways predicted by Correlation AnalyzeR software. **Fig. S3.** TEM1 is correlated with TGF-β related genes predicted by GRNdb software. **Fig. S4.** Cellular heterogeneity in normal scars and keloids is identified at single cell level. **Fig. S5. **Biological functions in each fibroblast subset of normal scars and keloids are annotated by GO database. **Fig. S6. **Cellular heterogeneity in normal skin and hypertrophic scars is identified at single cell level. **Fig. S7.** TEM1 protein is specifically expressed in primary dermal fibroblasts. **Fig. S8.** Proliferation, migration, and invasion are enhanced in keloid fibroblasts as compared with normal fibroblasts. **Fig. S9.** The effect of TEM1 on mouse skin fibroblast cell migration and SMAD2 nuclear translocation**. Fig. S10.** The effect of TEM1 on cell migration and nuclear translocation of SMAD2 in HEK293 cell line. **Fig. S11. **Effect of ontuxizumab on keloid size and collagen density in a xenograft nude mouse model. **Fig. S12.** TEM1 is essential for PDGF-mediated activity in keloid fibroblasts. **Table S1.** Patient data. **Table S2.** Primers for **RT-qPCR. Table S3.** Antibodies. **Table S4. **Pathway analysis of gene ontology in differentially expressed genes of cluster 1 in Fig. 4D

## Data Availability

All data associated with this study are present in the paper or in the Supporting Information. The scRNA-seq data of keloid from the published paper are available in the Gene Expression Omnibus (GEO) under accession GSE163973 [21]. The RNA-seq data of scar tissue in a mouse model of traction-induced hypertrophic scar have been deposited in the Gene Expression Omnibus (GEO) under accession GSE207284. Data, codes, and materials will be made available upon request.

## Abbreviations

ECM: Extracellular matrix
NF: Normal fibroblasts
KF: Keloid fibroblasts
TGF-β1: Transforming growth factor-β1
TGFBR1 and TGFBR2: TGF-β receptor I/II
TEM1: Tumor endothelial marker 1
Nskin: Normal skin
Nscar: Normal scar
Hscar: Hypertrophic scar
Kscar: Keloid scar
sNskin: Surrounding normal skin of keloid
α-SMA: α-Smooth muscle actin
COL1A1: Collagen type I alpha 1 chain
FN1: Fibronectin
DEGs: Differentially expressed genes
IPA: Ingenuity pathway analysis
GO: Gene ontology
