# Supplementary material for: TEM1/endosialin/CD248 promotes pathologic scarring and TGF-β activity through its receptor stability in dermal fibroblasts
Source: J Biomed Sci. 2024 Jan 23;31:12. doi: 10.1186/s12929-024-01001-0 (PMC10804696; doi:10.1186/s12929-024-01001-0)
Supplement: Supplementary file 1 — Additional file 1: Fig. S1. TEM1 is upregulated in the tissues and fibroblasts of keloids. Fig. S2. TEM1 is correlated with TGF-β pathways predicted by Correlation AnalyzeR software. Fig. S3. TEM1 is correlated with TGF-β related genes predicted by GRNdb software. Fig. S4. Cellular heterogeneity in normal scars and keloids is identified at single cell level. Fig. S5. Biological functions in each fibroblast subset of normal scars and keloids are annotated by GO database. Fig. S6. Cellular heterogeneity in normal skin and hypertrophic scars is identified at single cell level. Fig. S7. TEM1 protein is specifically expressed in primary dermal fibroblasts. Fig. S8. Proliferation, migration, and invasion are enhanced in keloid fibroblasts as compared with normal fibroblasts. Fig. S9. The effect of TEM1 on mouse skin fibroblast cell migration and SMAD2 nuclear translocation. Fig. S10. The effect of TEM1 on cell migration and nuclear translocation of SMAD2 in HEK293 cell line. Fig. S11. Effect of ontuxizumab on keloid size and collagen density in a xenograft nude mouse model. Fig. S12. TEM1 is essential for PDGF-mediated activity in keloid fibroblasts. Table S1. Patient data. Table S2. Primers for RT-qPCR. Table S3. Antibodies. Table S4. Pathway analysis of gene ontology in differentially expressed genes of cluster 1 in Fig. 4D [file 12929_2024_1001_MOESM1_ESM.docx]

**Supporting Information for**

**TEM1/endosialin/CD248 promotes pathologic scarring and TGF-β activity through its receptor stability in dermal fibroblasts**

Yi-Kai Hong, Yu-Chen Lin, Tsung-Lin Cheng, Chao-Han Lai, Yi-Han Chang, Yu-Lun Huang, Chia-Yi Hung, Chen-Han Wu, Kuo-Shu Hung, Ya-Chu Ku, Yen-Ting Ho, Ming-Jer Tang, Shu-Wha Lin, Guey-Yueh Shi, John A McGrath, Hua-Lin Wu, and Chao-Kai Hsu

Corresponding authors:

Hua-Lin Wu, halnwu@mail.ncku.edu.tw

Chao-Kai Hsu, kylehsu@mail.ncku.edu.tw

**This file includes:**

Figures S1 to S12

Tables S1 to S4

**Supplementary reference**

1. Hsu C.K., Lin H.H., Harn H.I., Ogawa R., Wang Y.K., Ho Y.T., Chen W.R., Lee Y.C., Lee J.Y., Shieh S.J., Cheng C.M., McGrath J.A. and Tang M.J. Caveolin-1 Controls Hyperresponsiveness to Mechanical Stimuli and Fibrogenesis-Associated RUNX2 Activation in Keloid Fibroblasts. J Invest Dermatol. 138(1):208-218, 2018.

**Supplementary figures**

**
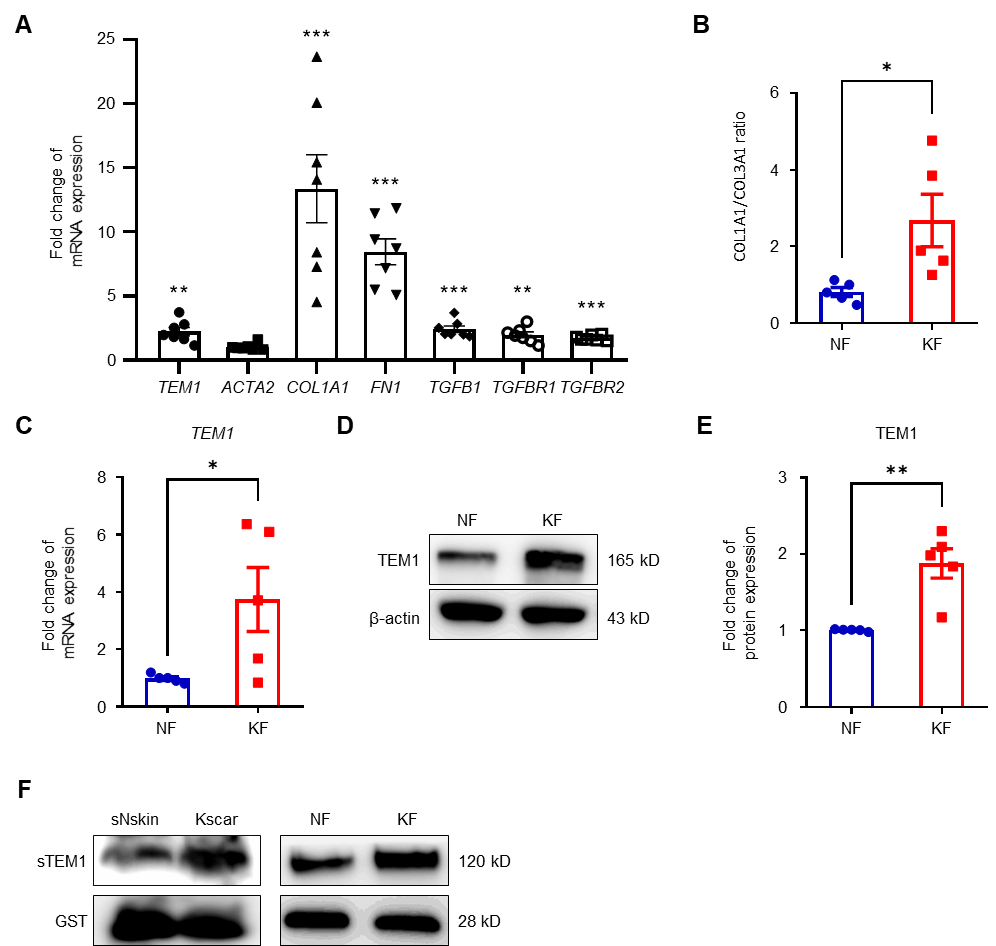
**

**Fig. S1 TEM1 is upregulated in the tissues and fibroblasts of keloids. A** Re-analyses of microarray data from our previous publication [[1](#_ENREF_1)] reveal the relative fold change of *TEM1*, *ACTA2*, *COL1A1*, *FN1*, *TGFB1*, *TGFBR1*, *TGFBR2* gene expression in Kscar (n = 7), compared with that in sNscar (n = 7). **B** The mRNA levels of *COL1A1* and *COL3A1* from the cell lysates of normal fibroblasts (NFs) (n = 4) and keloid fibroblasts (KFs) (n = 4) are detected using real-time PCR and then neutralized by *ACTB* gene. The ratio of *COL1A1* to *COL3A1* is shown. **C** The mRNA level of *TEM1* expression is examined with real-time PCR and then neutralized by *ACTB* gene. The fold change of *TEM1* mRNA expression in KFs (n = 4) is calculated with reference to NFs (n = 4). **D** The protein levels of TEM1 expression in NFs (n = 5) and KFs (n = 5) are assayed by Western blotting. **E** The intensity of each protein expression relative to ACTB is calculated based on the results of Western blotting. The fold change of TEM1 protein expression in KFs is determined as compared with NFs. **F** The conditioned medium harvested from tissues and fibroblasts of sNskin and Kscar was analyzed for TEM1 expression using Western blotting. Bar graphs show mean ± SEM. * *P* < 0.05, ** *P* < 0.01, *** *P* < 0.001. *P*-values are determined by an unpaired two-tailed Student’s *t*-test.

**
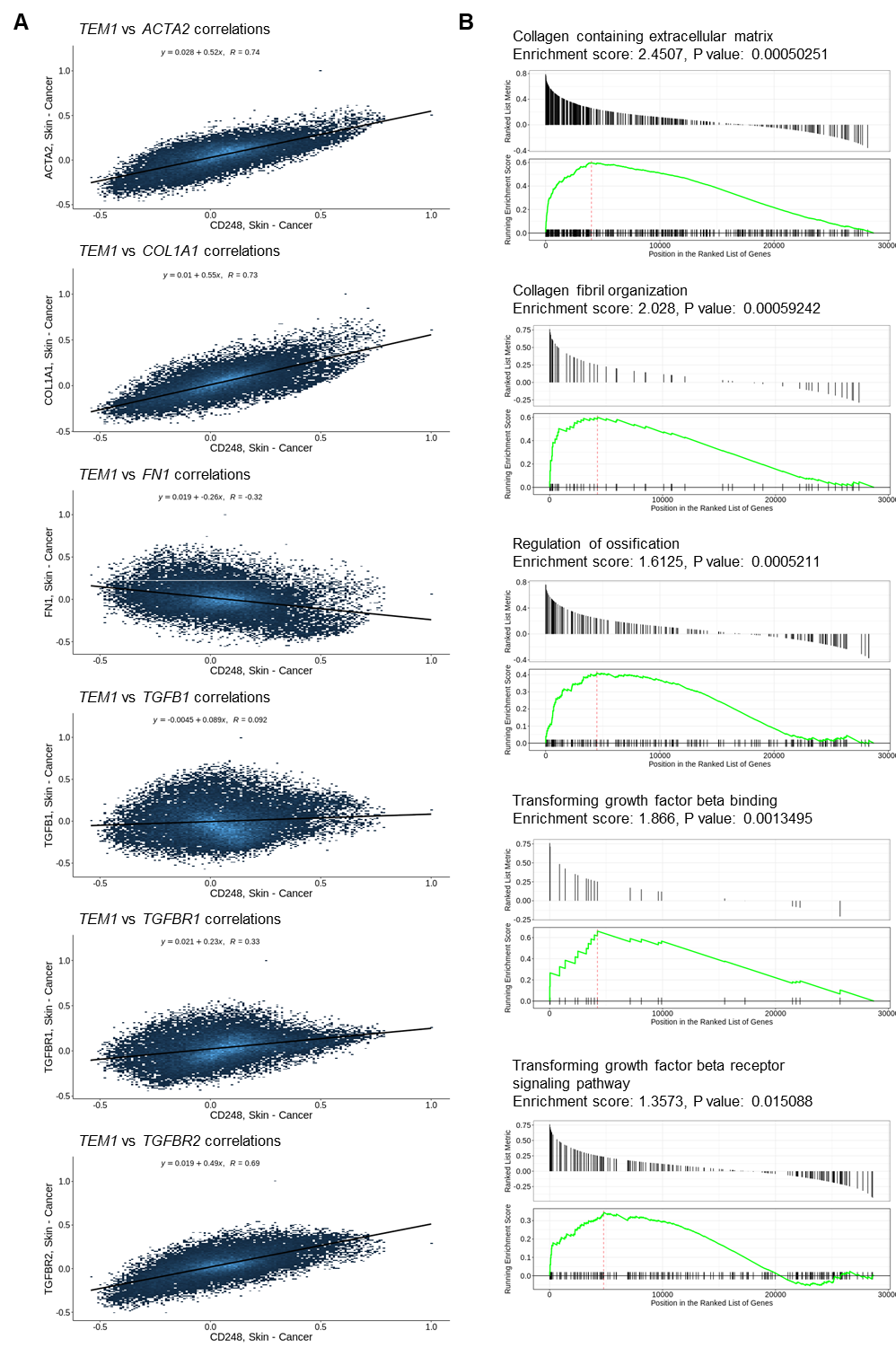
**

**Fig. S2 TEM1 is correlated with TGF-β pathways predicted by Correlation AnalyzeR software. A** The co-expression correlation of *TEM1* with *ACTA2*, *COL1A1*, *FN1*, *TGFB1*, *TGFBR1*, and *TGFBR2* is analyzed by Correlation AnalyzeR software in human skin samples. **B** Correlated gene set enrichment of *TEM1* gene is also analyzed by Correlation AnalyzeR software in human skin samples.


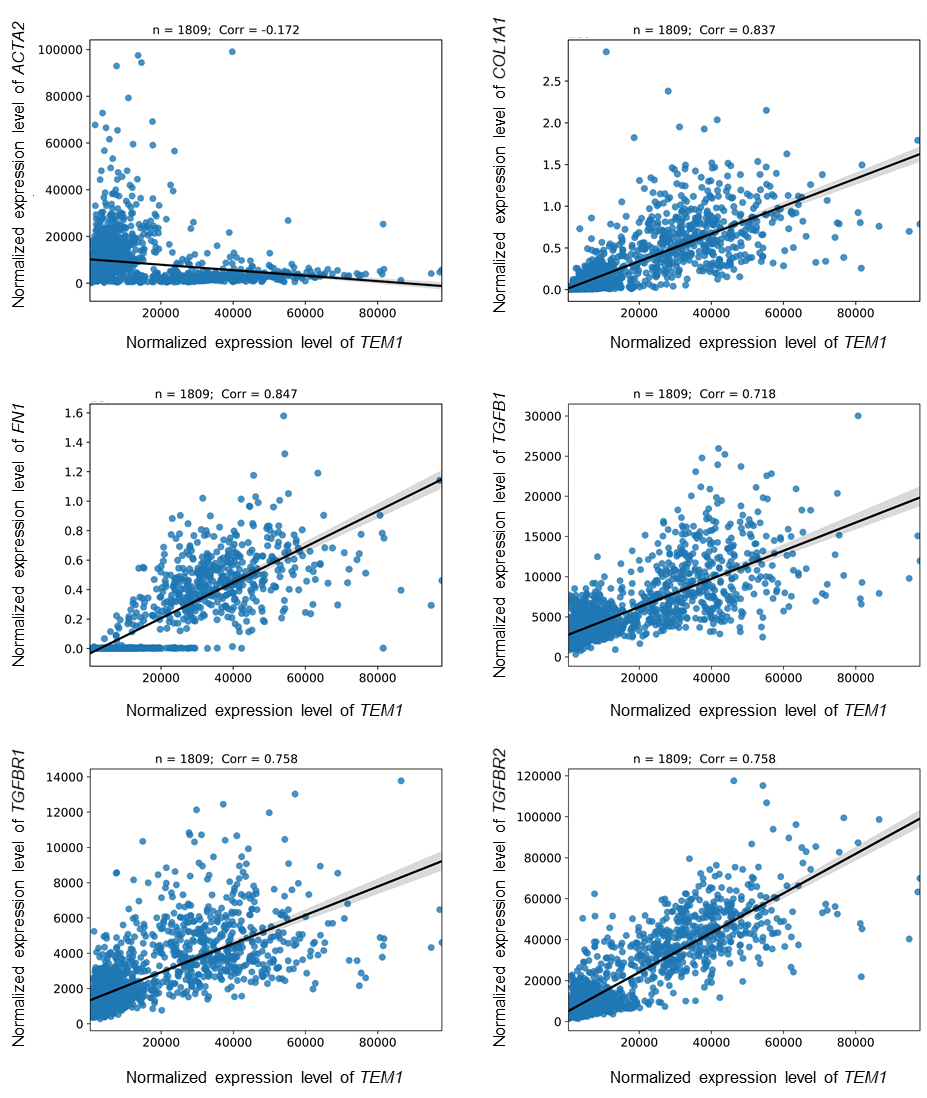


**Fig. S3 TEM1 is correlated with TGF-β related genes predicted by GRNdb software.** The co-expression correlation of *TEM1* with *ACTA2*, *COL1A1*, *FN1*, *TGFB1*, *TGFBR1*, and *TGFBR2* is analyzed by GRNdb software in human skin samples.

**
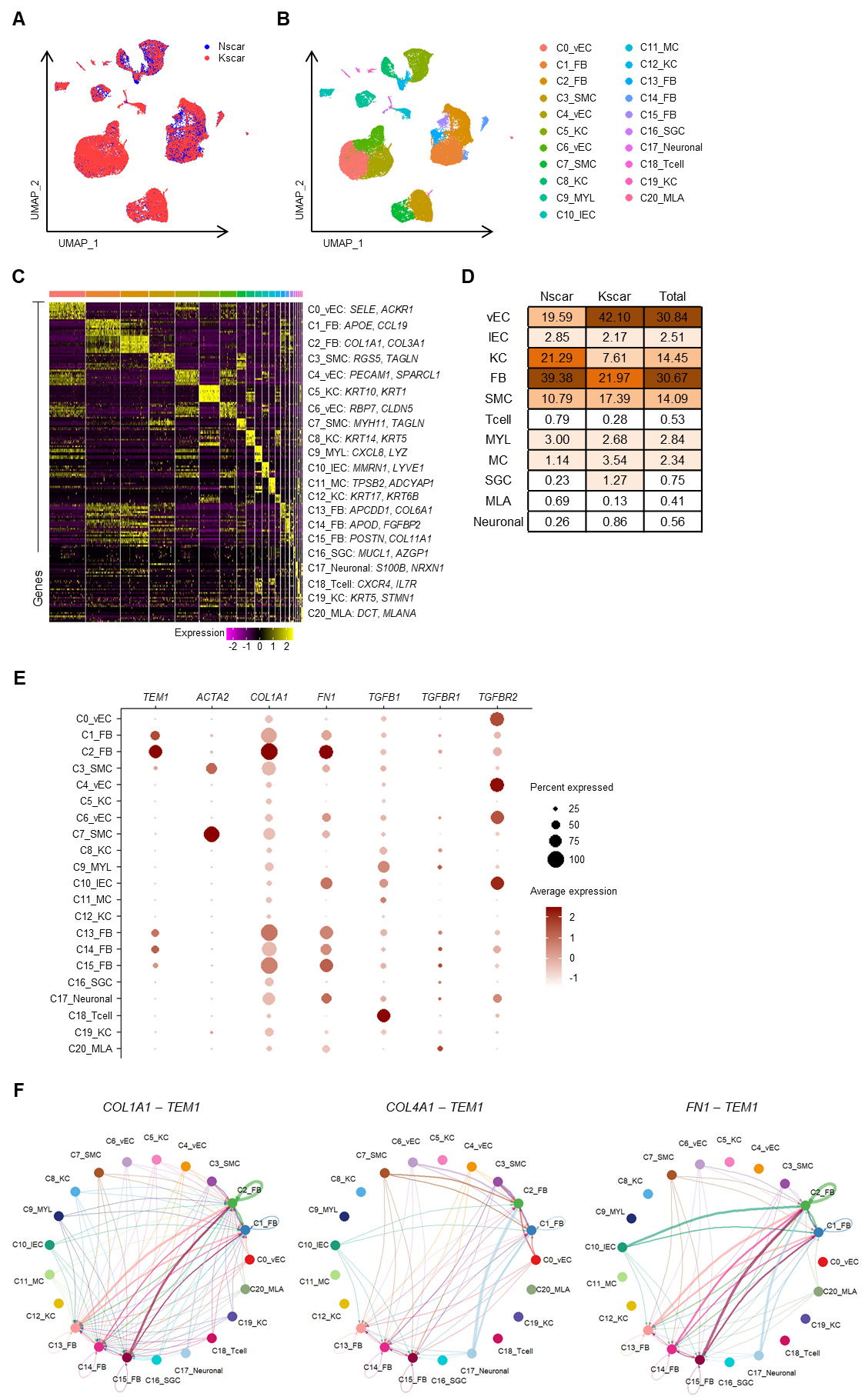
**

**Fig. S4 Cellular heterogeneity in normal scars and keloids is identified at single cell level. A, B** Uniform manifold approximation and projection (UMAP) plot reveals the integrated result of normal scars and keloids (**A**), and cellular heterogeneity with 21 distinct cell clusters (**B**). General identity is labeled on the right side. **C** Heatmap shows the top 10 differentially expressed genes (DEGs) in each cell type. Selected genes for each cluster are shown on the right side. **D** The percentages of each cell type in normal scars and keloids are present. **E** A set of distinctly expressed genes in all populations are shown by dot plots. **F** Circle plots illustrating autocrine and paracrine signaling interactions of ligand-receptor pairs: COL1A1-TEM1, COL4A1-TEM1, and FN1-TEM1 across various cell populations.

**
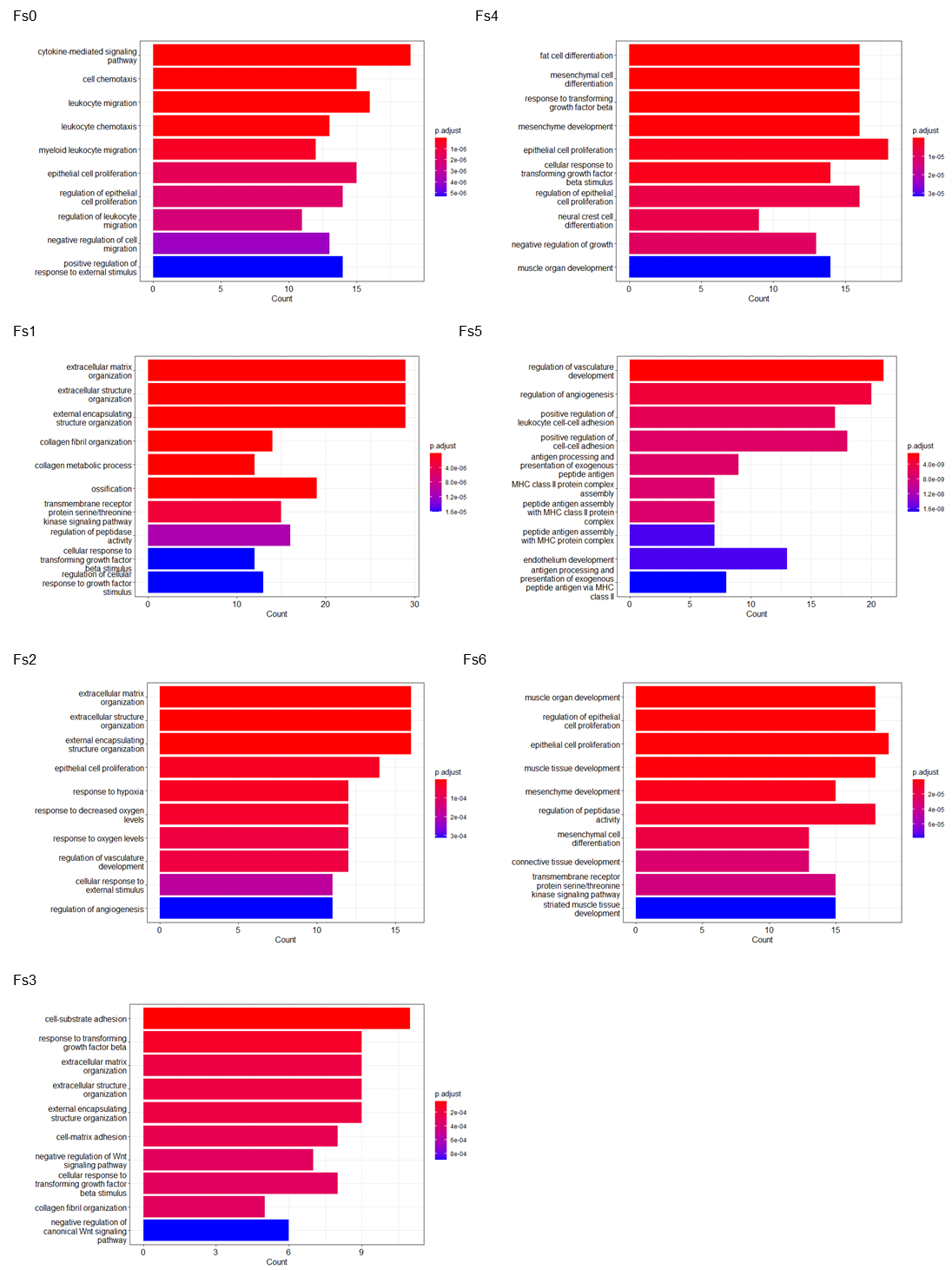
**

**Fig. S5 Biological functions in each fibroblast subset of normal scars and keloids are annotated by GO database.** The GO biological processes of DEGs in each fibroblast subgroup are analyzed by clusterProfiler software.

**
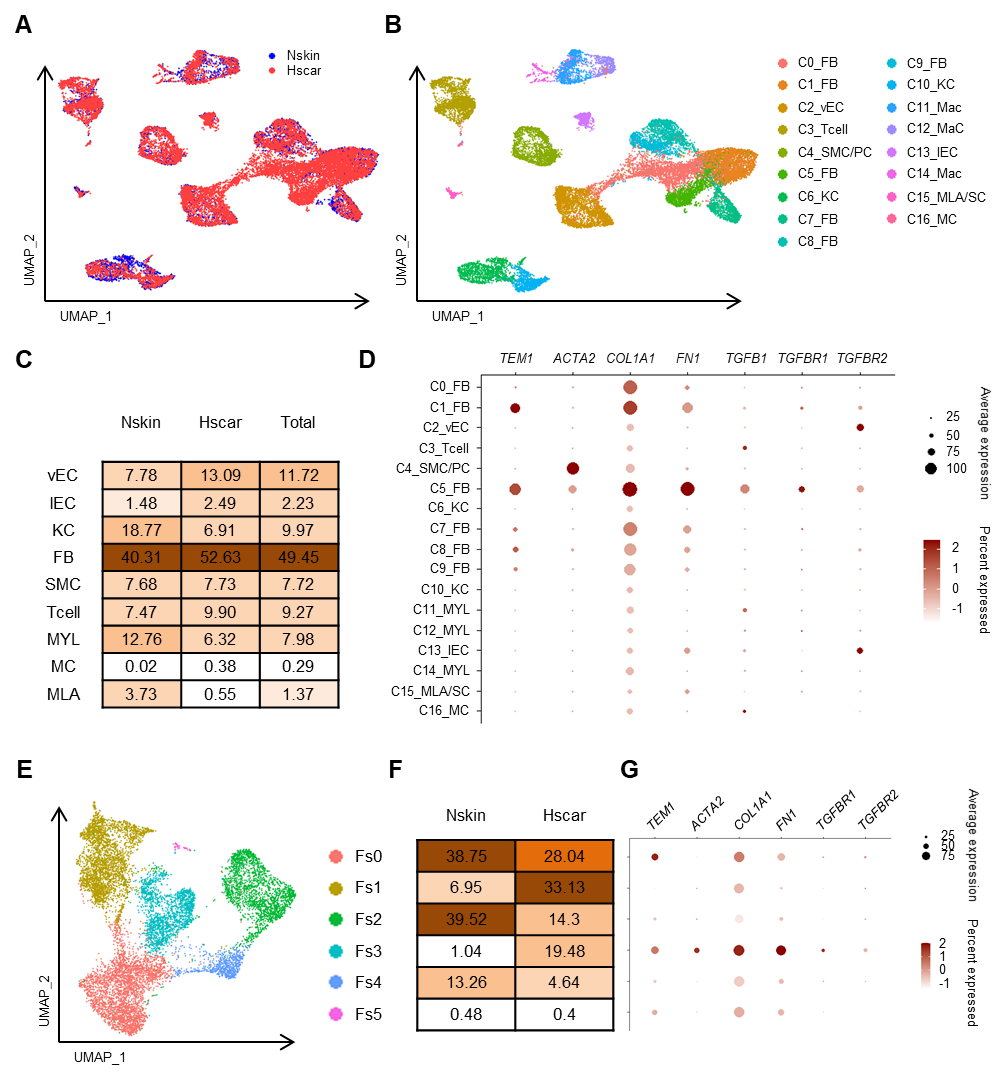
**

**Fig. S6 Cellular heterogeneity in normal skin and hypertrophic scars is identified at single cell level. A, B** Uniform manifold approximation and projection (UMAP) plot reveals the integrated result of normal skin and hypertrophic scar (**A**), and cellular heterogeneity with 17 distinct cell clusters (**B**). General identity is labeled on the right side. **C** The percentages of each cell type in normal scars and keloids are present. **D** A set of distinctly expressed genes in all populations are shown by dot plots. **E** The subclusters of all ﬁbroblasts from normal scars and keloids are further classified into 6 distinct subtypes (Fs0–Fs5). **F** Percentages of each fibroblast subgroup in normal scars and keloids are presented. **G** Sets of distinctly expressed genes in all fibroblast subsets are shown by dot plots.

**
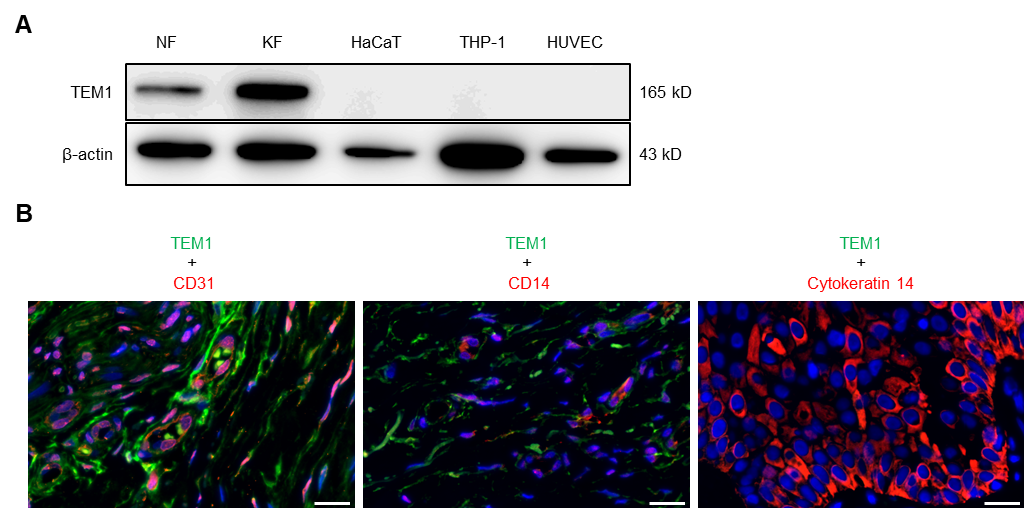
**

**Fig. S7 TEM1 protein is specifically expressed in primary dermal fibroblasts. A** The expression of TEM1 and β-actin proteins in the lysate of different cell types including NF, KF, HaCaT, THP-1, and HUVEC lines is determined by Western blotting. **B** The tissue sections from keloid scar are immunostained for TEM1, CD31, CD14, and cytokeratin 14. The nucleus is stained with DAPI. Scale bar = 20 μm.

**
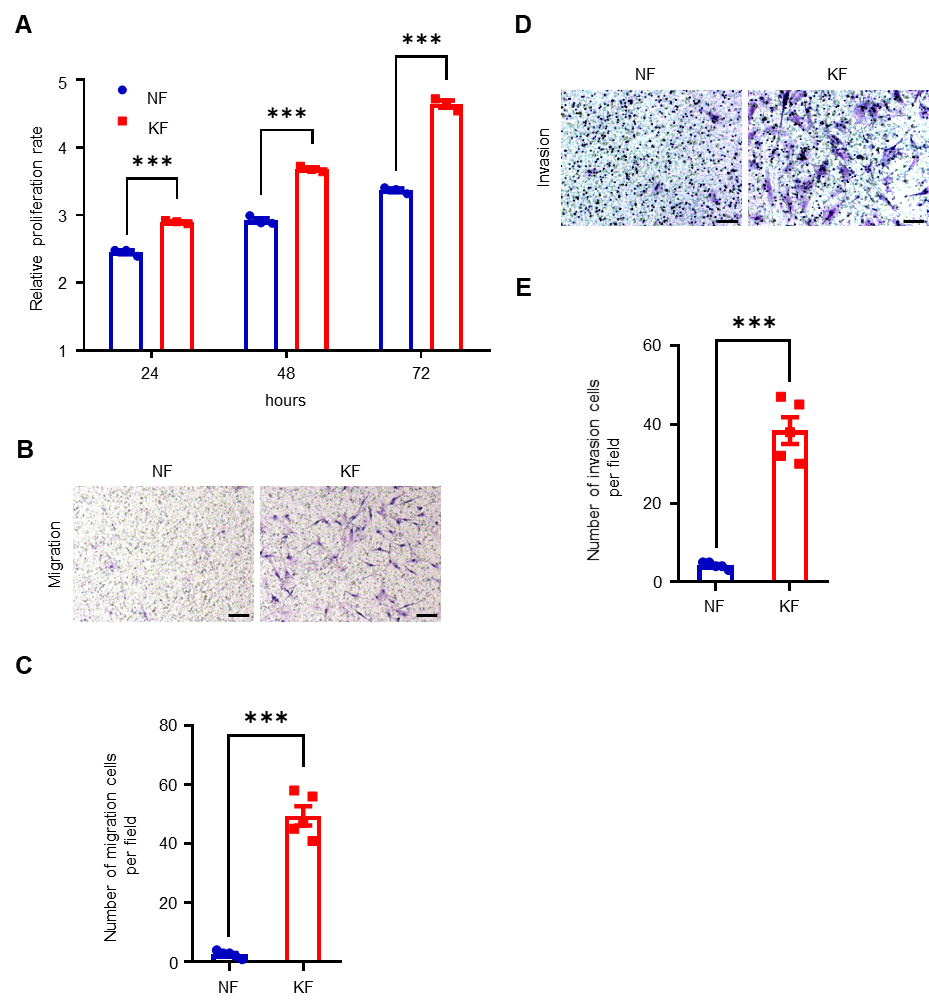
**

**Fig. S8 Proliferation, migration, and invasion are enhanced in keloid fibroblasts as compared with normal fibroblasts. A** The cell viability of fibroblasts in 10% FBS is measured by WST-1 assay. Relative proliferation of 24, 48, and 72 hours to 0 hour is shown. **B, C** Cell migration of fibroblasts is measured by transwell migration assay with 10% FBS as chemoattractant. Scale bar = 100 μm. **D, E** Cell invasion of fibroblasts is assayed using matrigel-coated transwell migration assay with 10% FBS as chemoattractant. Scale bar = 100 μm. Bar graphs show mean ± SEM. *** *P* < 0.001. *P*-values are determined by unpaired two-tailed Student’s *t*-test and two-way analysis of variance.

**
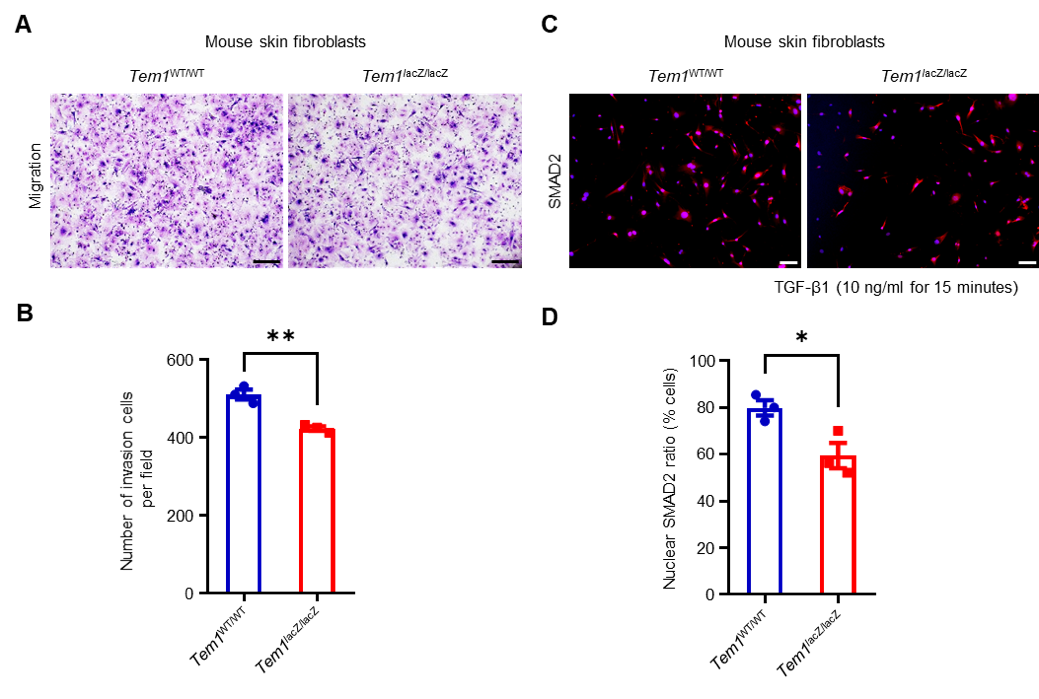
**

**Fig. S9 The effect of TEM1 on mouse skin fibroblast cell migration and SMAD2 nuclear translocation.** **A, B** Transwell migration assay showcasing the migratory capabilities of mouse skin fibroblasts derived from *Tem1*^wt/wt^ and *Tem1*^lacZ/wt^ mice, using 10% FBS as the chemoattractant. Scale bar = 100 μm. **C, D** Immunofluorescence study of SMAD2 nuclear translocation in mouse skin fibroblasts following treatment with TGF-β1 at a concentration of 10 ng/ml. Quantification of the nuclear SMAD2 to DAPI ratio was performed using ImageJ. Scale bar = 100 μm.

**
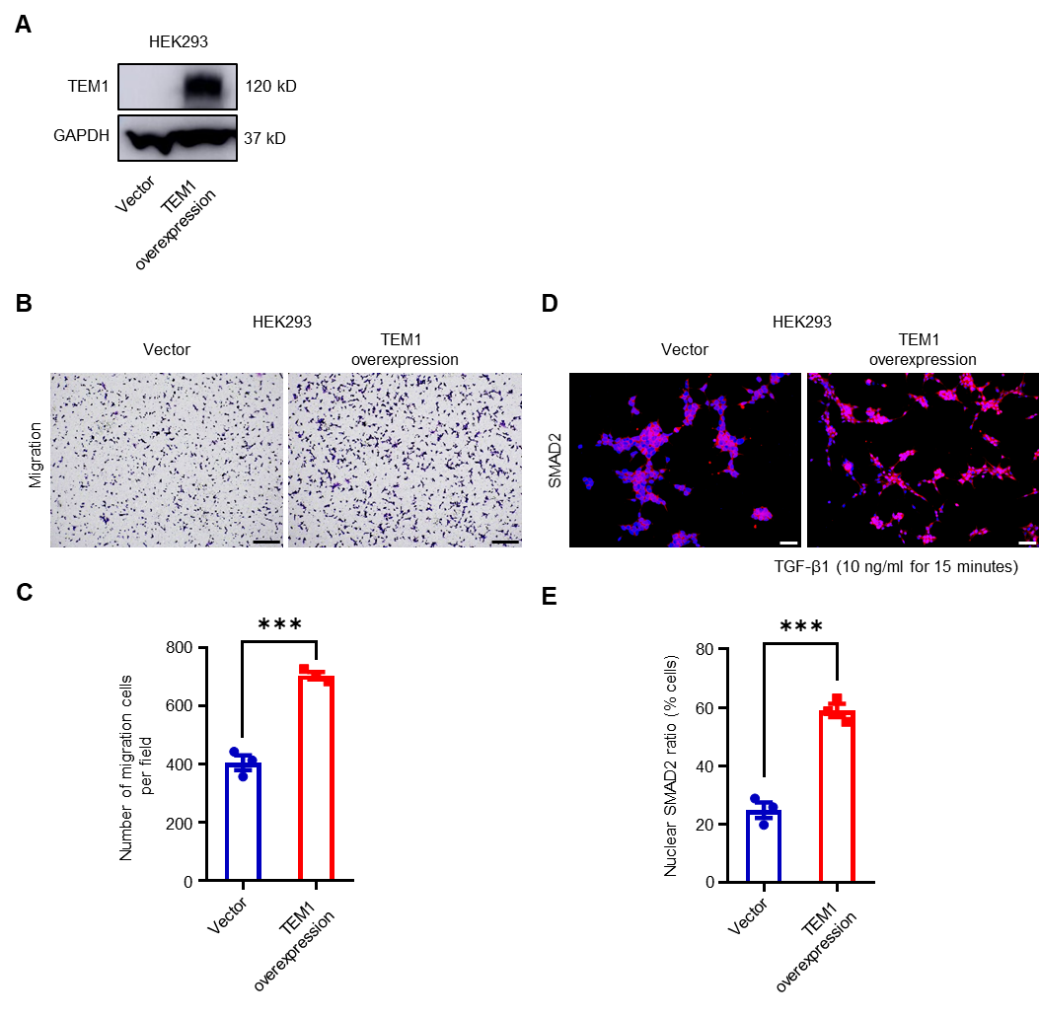
**

**Fig. S10 The effect of TEM1 on cell migration and nuclear translocation of SMAD2 in HEK293 cell line. A** Establishment of a TEM1-overexpressing HEK293 cell line via transfection using pEGFP-hTEM1, with pEGFP-N1 serving as the control vector. TEM1 protein expression levels are evaluated using Western blotting. **B, C** Assessment of HEK293 cell migration capabilities using a transwell migration assay, with 10% FBS acting as the chemoattractant. Scale bar = 100 μm. **D, E** Immunofluorescence study of SMAD2 nuclear translocation in HEK293 cells post-treatment with TGF-β1 at 10 ng/ml concentration. The proportion of nuclear SMAD2 relative to DAPI is quantitatively analyzed using ImageJ. Scale bar = 100 μm.


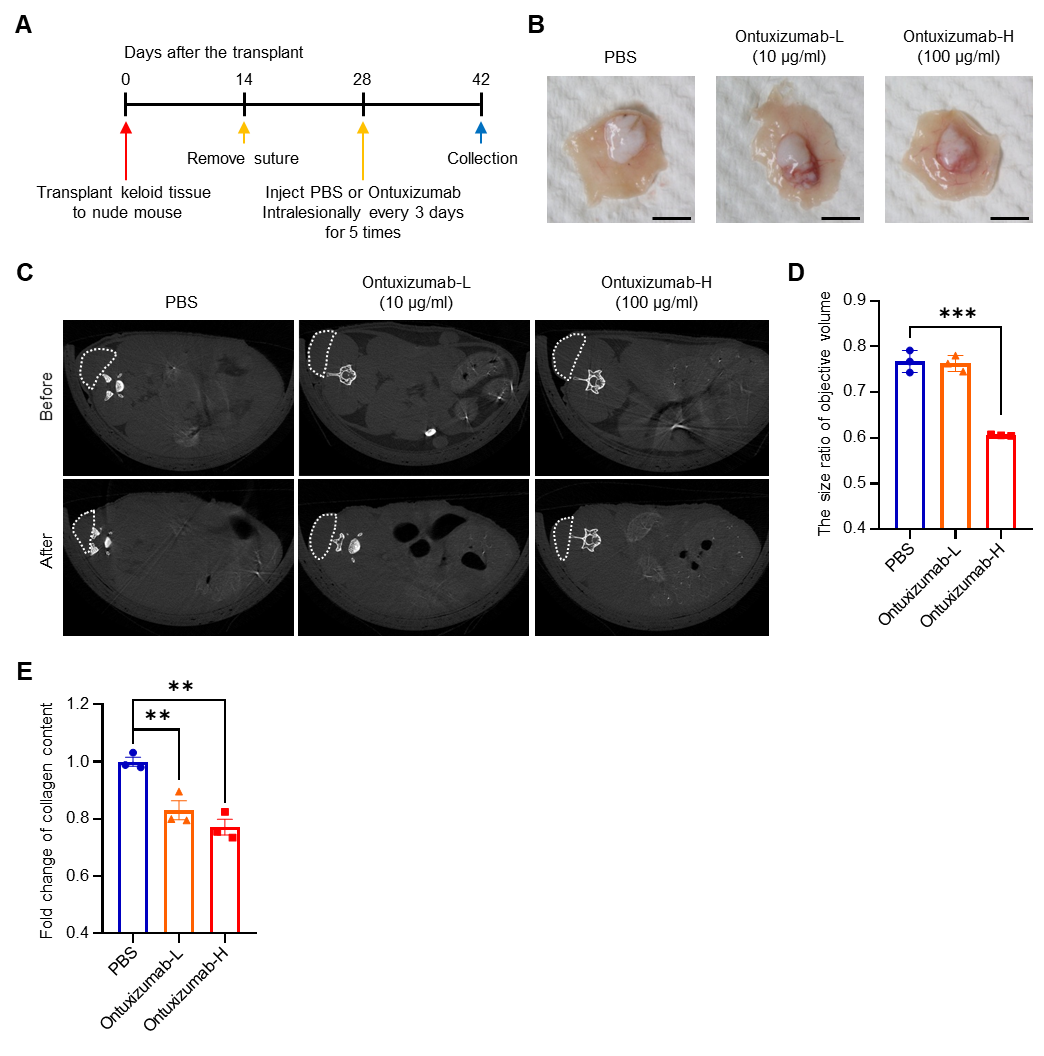


**Fig. S11 Effect of ontuxizumab on keloid size and collagen density in a xenograft nude mouse model. A** Experimental timeline detailing the steps in the keloid xenograft nude mouse model, from transplantation of keloid tissue to collection post-treatment. **B** Morphological observation of keloid tissues from xenografted nude mice after local injection with either PBS, low-dose ontuxizumab (10 μg/ml), or high-dose ontuxizumab (100 μg/ml), visualized using a dissecting microscope. Scale bar = 5 mm. **C** Micro-computed tomography images highlighting the keloid lesions before and after ontuxizumab treatment. **D** Quantification of the size ratio of keloid volume post-treatment in comparison to the volume before treatment, which is used as a reference. **E** Analysis of collagen density in excised keloid tissues. Tissues were stained with picrosirius red and subsequently quantified using ImageJ in a blinded manner.


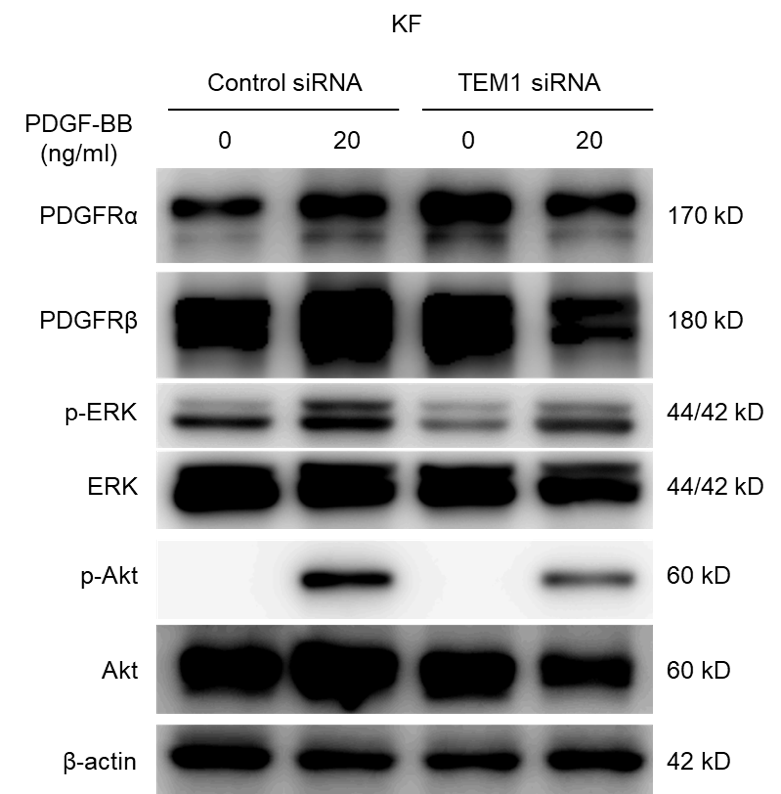


**Fig. S12 TEM1 is essential for PDGF-mediated activity in keloid fibroblasts.** The amount of protein expression, including PDGFRα, PDGFRβ, p-ERK, ERK, p-Akt, and β-actin, in KFs treated with PDGF-BB (20 ng/ml) for 15 minutes is analyzed using Western blotting.

**Supplementary tables**

**Table S1. Patient data**

| Cell type | Age (years) | Sex | Biopsy site | Duration (years) |
| --- | --- | --- | --- | --- |
| Keloid |  |  |  |  |
| K-1 | 23 | Female | Ear | 3 |
| K-2 | 27 | Male | Chest | 12 |
| K-3 | 23 | Male | Ear | 2 |
| K-4 | 28 | Male | Ear | 1.5 |
| K-5 | 21 | Female | Ear | 2 |
| K-6 | 25 | Male | Shoulder | 5 |
| K-7 | 29 | Male | Chest | 14 |
| K-8 | 57 | Female | Chest | 10 |
| Control |  |  |  |  |
| N-1 | 34 | Male | Back | -- |
| N-2 | 37 | Male | Forearm | -- |
| N-3 | 19 | Female | Abdomen | -- |
| N-4 | 22 | Female | Upper arm | -- |
| N-5 | 36 | Female | Back | -- |
| N-6 | 28 | Male | Buttock | -- |
| N-7 | 39 | Female | Abdomen | -- |
| N-8 | 39 | Male | Shoulder | -- |

**Table S2. Primers for RT-qPCR**

| Gene | Species | Primer type | Seqence |
| --- | --- | --- | --- |
| *CD248* | human | Forward | AGTGTTATTGTAGCGAGGGACA |
|  |  | Reverse | CCTCTGGGAAGCTCGGTCTA |
| *GAPDH* | human | Forward | AGGTCATCCCTGAGCTGAACGG |
|  |  | Reverse | CGCCTGCTTCACCACCTTCTTG |
| *COL1A1* | human | Forward | GAGGGCCAAGACGAAGACATC |
|  |  | Reverse | CAGATCACGTCATCGCACAAC |
| *COL3A1* | human | Forward | GGAGCTGGCTACTTCTCGC |
|  |  | Reverse | GGGAACATCCTCCTTCAACAG |
| *TGFBR1* | human | Forward | CACAGAGTGGGAACAAAAAGGT |
|  |  | Reverse | CCAATGGAACATCGTCGAGCA |
| *TGFBR2* | human | Forward | AAGATGACCGCTCTGACATCA |
|  |  | Reverse | CTTATAGACCTCAGCAAAGCGA |
| *Col1a1* | mouse | Forward | CAGTCGATTCACCTACAGCACG |
|  |  | Reverse | GGGATGGAGGGAGTTTACACG |
| *Col3a1* | mouse | Forward | GTTCTAGAGGATGGCTGTACTAAACACA |
|  |  | Reverse | TTGCCTTGCGTGTTTGATATTC |
| *Fn1* | mouse | Forward | TGCACAACCAATGAAGGGG |
|  |  | Reverse | ATCTCGAAGCTGCGAGTAGG |
| *Acta2* | mouse | Forward | GCGGGCATCCACGAAACCACCTAT |
|  |  | Reverse | GCTTTGGGCAGGAATGATTTGGAA |
|  |  |  |  |

**Table S3. Antibodies**

| Primary antibody,  Company (Product catalog number) | Primary Ab raised species, isotype, concentration | MW  (kD) | Secondary Ab Concentration | |
| --- | --- | --- | --- | --- |
|  |  |  | WB | IHC/ICC |
| TEM1/CD248  Proteintech (18160-1-AP) | Rabbit, IgG  (WB 1:4000; ICC 1:100) | 165 | 1:4000 | 1:200 |
| TEM1/CD248  Atlas Antibodies (HPA051856) | Rabbit, IgG  (ICC, IHC, IF 1:100) | - |  | 1:200 |
| GAPDH  Santa Cruz (sc-32233) | Mouse, IgG  (WB 1:5000) | 37 | 1:4000 |  |
| β-actin  Santa Cruz (sc-47778) | Mouse, IgG  (WB 1:2000) | 43 | 1:4000 |  |
| p-ERK  Santa Cruz (sc-7383) | Mouse, IgG  (WB 1:1000) | 42/44 | 1:4000 |  |
| ERK  Santa Cruz (sc-94) | Rabbit, IgG  (WB 1:10000) | 42/44 | 1:4000 |  |
| p- SMAD2  Cell signaling (8828S) | Rabbit, IgG  (WB 1:1000) | 60 | 1:4000 |  |
| SMAD2/3  Santa Cruz (sc-133098) | Mouse, IgG  (WB 1:1000; ICC 1:50) | 60 | 1:4000 | 1:200 |
| COL1A1  Santa Cruz (sc-293182) | Mouse, IgG  (IF 1:20) |  |  | 1:200 |
| FN1  Santa Cruz (sc-8422) | Mouse, IgG  (WB 1:1000; IF 1:20) | 210 | 1:4000 | 1:200 |
| α-SMA  Sigma (A2547) | Mouse, IgG  (IF 1:100) |  |  | 1:200 |
| Ubiquitin  Cell signaling (#3936) | Mouse, IgG  (WB 1:5000) | 220 | 1:4000 |  |
| Cyclin D1  Cell signaling (#2978) | Rabbit, IgG  (WB 1:5000) | 36 | 1:4000 |  |
| α-SMA  Abcam (ab5694) | Rabbit, IgG  (WB 1:5000) | 42 | 1:4000 | 1:200 |
| COL1A1  Santa Cruz (sc-8784R) | Rabbit, IgG  (WB 1:2000) |  | 1:4000 | 1:200 |
| TGFBR1  Santa Cruz (sc-101574) | Mouse, IgG  (WB 1:200) | 54 | 1:4000 |  |
| TGFBR2  Santa Cruz (sc-17792) | Mouse, IgG  (WB 1:200, IF 1:20, ICC 1:20) | 63-95 | 1:4000 | 1:200 |
| CD31  Santa Cruz (sc-1506) | Goat, IgG  (IF 1:20) |  |  | 1:200 |
| CD14  Santa Cruz (sc-1182) | Mouse, IgG  (IF 1:20) |  |  | 1:200 |
| Cytokeratin 14  Abcam (ab7800) | Mouse, IgG  (IF 1:200) |  |  | 1:200 |

**Table S4. Pathway analysis of gene ontology in differentially expressed genes of cluster 1 in Fig. 4D**

| Ranking | ID | Description | -LOG_10_ *P*-value |
| --- | --- | --- | --- |
| 1 | GO:0030198 | extracellular matrix organization | 28.76406 |
| 2 | GO:0043062 | extracellular structure organization | 28.66811 |
| 3 | GO:0031589 | cell-substrate adhesion | 23.08732 |
| 4 | GO:0001667 | ameboidal-type cell migration | 21.27629 |
| 5 | GO:0009100 | glycoprotein metabolic process | 20.96941 |
| 6 | GO:1901342 | regulation of vasculature development | 19.97379 |
| 7 | GO:0009101 | glycoprotein biosynthetic process | 18.29928 |
| 8 | GO:0090130 | tissue migration | 17.80523 |
| 9 | GO:0045765 | regulation of angiogenesis | 17.71907 |
| 10 | GO:0010631 | epithelial cell migration | 17.40397 |
| 11 | GO:0090132 | epithelium migration | 17.26612 |
| 12 | GO:0007264 | small GTPase mediated signal transduction | 16.42769 |
| 13 | GO:0007160 | cell-matrix adhesion | 16.40896 |
| 14 | GO:0070085 | glycosylation | 15.42294 |
| 15 | GO:0006486 | protein glycosylation | 15.16995 |
| 16 | GO:0043413 | macromolecule glycosylation | 15.16995 |
| 17 | GO:0043542 | endothelial cell migration | 15.10688 |
| 18 | GO:0045785 | positive regulation of cell adhesion | 14.82794 |
| 19 | GO:0030199 | collagen fibril organization | 14.4059 |
| 20 | GO:0032963 | collagen metabolic process | 14.24041 |
| 21 | GO:0007265 | Ras protein signal transduction | 14.04269 |
| 22 | GO:0071560 | cellular response to transforming growth factor beta stimulus | 13.98634 |
| 23 | GO:0034446 | substrate adhesion-dependent cell spreading | 13.94003 |
| 24 | GO:0001655 | urogenital system development | 13.83584 |
| 25 | GO:0090287 | regulation of cellular response to growth factor stimulus | 13.73381 |
| 26 | GO:0071559 | response to transforming growth factor beta | 13.69091 |
| 27 | GO:0034329 | cell junction assembly | 13.6125 |
| 28 | GO:0007229 | integrin-mediated signaling pathway | 13.47118 |
| 29 | GO:0010632 | regulation of epithelial cell migration | 13.44067 |
| 30 | GO:0051271 | negative regulation of cellular component movement | 12.98626 |
| 75 | GO:0016055 | Wnt signaling pathway | 9.415286 |
| 170 | GO:0008277 | regulation of G protein-coupled receptor signaling pathway | 6.139243 |
| 199 | GO:0035791 | platelet-derived growth factor receptor-beta signaling pathway | 5.819267 |
| 209 | GO:0035924 | cellular response to vascular endothelial growth factor stimulus | 5.711744 |
| 347 | GO:0044344 | cellular response to fibroblast growth factor stimulus | 4.548023 |
| 442 | GO:0007173 | epidermal growth factor receptor signaling pathway | 3.910373 |
